# Photo-affinity labelling and biochemical analyses identify the target of trypanocidal simplified natural product analogues

**DOI:** 10.1371/journal.pntd.0005886

**Published:** 2017-09-05

**Authors:** Lindsay B. Tulloch, Stefanie K. Menzies, Andrew L. Fraser, Eoin R. Gould, Elizabeth F. King, Marija K. Zacharova, Gordon J. Florence, Terry K. Smith

**Affiliations:** EaStChem School of Chemistry and School of Biology, Biomedical Science Research Complex, University of St Andrews, St Andrews, Fife, United Kingdom; Instituto de Ciências Biológicas, Universidade Federal de Minas Gerais, BRAZIL

## Abstract

Current drugs to treat African sleeping sickness are inadequate and new therapies are urgently required. As part of a medicinal chemistry programme based upon the simplification of acetogenin-type ether scaffolds, we previously reported the promising trypanocidal activity of **compound 1**, a bis-tetrahydropyran 1,4-triazole (B-THP-T) inhibitor. This study aims to identify the protein target(s) of this class of compound in *Trypanosoma brucei* to understand its mode of action and aid further structural optimisation. We used **compound 3**, a diazirine- and alkyne-containing bi-functional photo-affinity probe analogue of our lead B-THP-T, **compound 1**, to identify potential targets of our lead compound in the procyclic form *T*. *brucei*. Bi-functional **compound 3** was UV cross-linked to its target(s) *in vivo* and biotin affinity or Cy5.5 reporter tags were subsequently appended by Cu(II)-catalysed azide-alkyne cycloaddition. The biotinylated protein adducts were isolated with streptavidin affinity beads and subsequent LC-MSMS identified the F_o_F_1_-ATP synthase (mitochondrial complex V) as a potential target. This target identification was confirmed using various different approaches. We show that (i) **compound 1** decreases cellular ATP levels (ii) by inhibiting oxidative phosphorylation (iii) at the F_o_F_1_-ATP synthase. Furthermore, the use of GFP-PTP-tagged subunits of the F_o_F_1_-ATP synthase, shows that our compounds bind specifically to both the α- and β-subunits of the ATP synthase. The F_o_F_1_-ATP synthase is a target of our simplified acetogenin-type analogues. This mitochondrial complex is essential in both procyclic and bloodstream forms of *T*. *brucei* and its identification as our target will enable further inhibitor optimisation towards future drug discovery. Furthermore, the photo-affinity labeling technique described here can be readily applied to other drugs of unknown targets to identify their modes of action and facilitate more broadly therapeutic drug design in any pathogen or disease model.

## Author summary

Millions of people are at risk of developing African sleeping sickness through infection with the parasite *Trypanosoma brucei*, which is fatal if untreated. Current therapies are antiquated and inadequate, requiring hospitalization to deliver them through complex regimens, thus new effective, cheap and easy to administer therapies are urgently required. We recently reported the generation of a new compound that is selectively active against the parasite responsible for the disease, but its mechanism of action was unknown. This body of work identifies the specific target within the parasite as the essential F_o_F_1_-ATP synthase (mitochondrial complex V), first by analysing the proteins bound to a tagged analogue of our active compound (in a process known as photo-affinity labelling), and secondly by determining the effects of our active compound on cellular metabolism. With the target now identified work can begin to improve inhibitor potency and selectivity with the aid of rational drug design and structural optimisation towards future drug discovery.

## Introduction

Protozoan parasites of the *Trypanosoma* genus cause widespread disease and death across large regions of the developing world. In sub-Saharan Africa *Trypanosoma brucei gambiense* and *T*. *b*. *rhodesiense* are the causative agents of human African trypanosomiasis (HAT, or African sleeping sickness) in humans while several species cause disease in livestock and wild animals, creating a major socio-economic burden to the African continent. The parasites are spread through the bites of infected tsetse flies and, if left untreated, infection is usually fatal. Over 65 million people who live in the tsetse fly habitat are at risk of infection and each year there are an estimated 15–20,000 new cases [1].

In the early 1900’s African trypanosomes became one of the first subjects of “modern drug discovery” when Paul Ehrlich, following his observations on differential cell stains, hypothesised that some molecules could be developed to target pathogens but not their hosts (a term he coined “chemotherapy”), and screened a library of synthetic dyes in trypanosome-infected animals to find a “magic bullet” [2,3]. Through a combination of rational synthetic chemistry and phenotypic screening his pioneering work led to the discoveries by others of suramin in 1917 and melarsoprol in 1949 [4], both of which are still front-line drugs for the treatment of early stage (suramin) and late stage (melarsprol) infection by *T*. *b*. *rhodesiense* [5]. Pentamindine, which is currently the first-line treatment for early stage infection by *T*. *b*. *gambiense* [5], was likewise developed from the anti-diabetic synthalin in 1937 [6,7]. However, HAT has been neglected over the past half century and all of these antiquated non-oral drugs are difficult to administer, are sometimes ineffective and are themselves toxic, often causing undesirable side effects with melarsoprol causing the death of up to 5% of those treated [5,8]. Furthermore, melarsoprol resistance is a growing issue [9–14] and new drugs are therefore urgently needed, particularly for late stage infection. Despite their antiquity and widespread use, the targets and modes of action of these currently used drugs are poorly understood, making it difficult to design to safer analogues. Investment from the pharmaceutical industry has been slow in forthcoming for this and related neglected diseases, which affect many of the poorest and most underdeveloped countries in the world, and efforts so far have been driven instead by charities and non-profit organisations.

**Fig 1:**
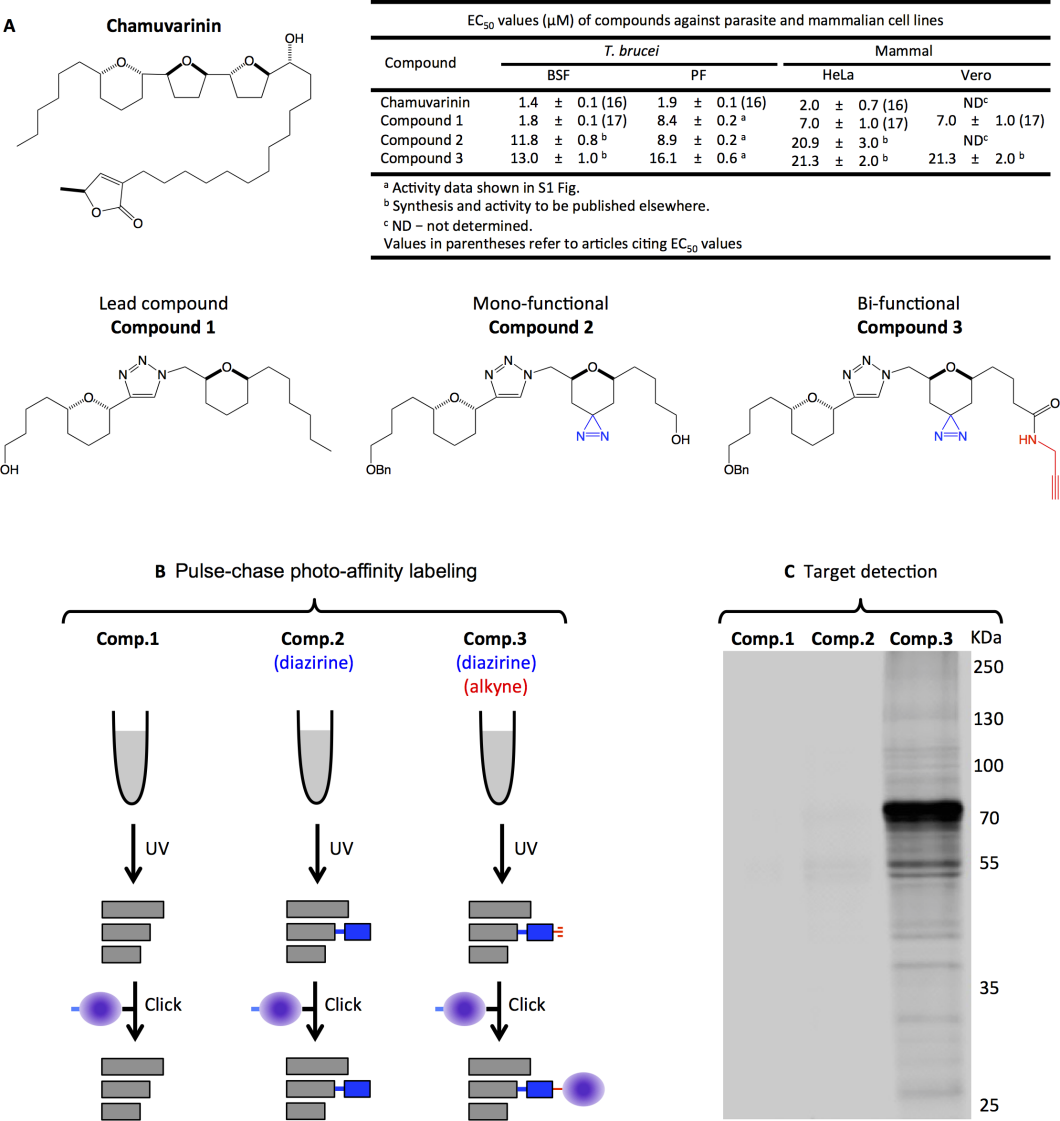
Labelling and detection of B-THP-T target(s). **(A)** Structures of chamuvarinin and simplified analogues thereof as probes used in this study and their respective toxicity in selected cell lines. Bi-functional **compound 3** contains a diazirine (blue) to cross-link the compound to its target and an alkyne handle (red) to which an azide-containing reporter probe can be added. Mono-functional **compound 2** contains the diazirine to cross-link to its target but lacks the alkyne handle and cannot be appended with an azide reporter probe. Lead B-THP-T, **compound 1**, lacks both diazirine and alkyne. The additional functional groups on **compounds 2** and **3** are minor alterations, which have little effect on inhibitor potency. **(B)** Overview of the *in vivo* pulse-chase photo-affinity labelling procedure. B-THP-T were incubated with live PF *T*. *brucei* cells and trafficked to their targets. Following UV-irradiation, only compounds containing a diazirine (i.e. **compounds 2** and **3**) could cross-link with target proteins. The subsequent “click reaction” appended the conjugated B-THP-T with a reporter probe as long as an alkyne handle was present (i.e. on **compound 3**). Refer to the experimental section for details. **(C)** Detection of Cy5.5-tagged proteins following photo-affinity labelling. Proteins were separated by SDS-PAGE and conjugated Cy5.5 was detected at 700 nm. Lanes 1–3 correspond to usage of compounds 1–3 respectively. Several proteins in lane 3 were conjugated with Cy5.5 via labelling with bi-functional compound 3. No Cy5.5 conjugation was detected in lanes 1 or 2 where the B-THP-T used lacked diazirine and/or alkyne handle, indicating that reporter labelling is specific to bi-functional compound 3.

Advances in automated liquid handling, cell culture and detection technology has allowed researchers and the pharmaceutical industry to return to phenotypic screening-based practices, as those pioneered by Ehrlich, for the latest drug discovery efforts. We recently reported the total synthesis and trypanocidal activity of the acetogenin, chamuvarinin [15,16] and non-natural bis-tetrahydropyran 1,4-triazole (B-THP-T) analogues thereof including **compound 1** ([17]; **Fig 1A**) using a phenotypic screening approach. Acetogenins are a family of over 400 structurally related fatty acid-derived natural products isolated from tropical plants of the *Annonaceae* family (for review, see [18]), and characteristically bear one to three tetrahydropyran (THP) and/or tetrahydrofuran (THF) rings flanked by a terminal γ-lactone head and a hydrophobic tail. Many members have been reported to display high inhibition of mitochondrial complex I [19–21], making them cytotoxic to a wide range of organisms [22,23], and their particularly high potency against ATP-hungry tumour cells (reviewed in [24]) has led to their investigation as potential anti-cancer chemotherapeutics; despite mammalian cells requiring complex I activity, pre-clinical trials with select acetogenins are encouraging, with some proving as effective and selective as Taxol, a first-line treatment for some cancers, at reducing solid tumours in mice [25]. Cytotoxic activities vary among acetogenins and between cell lines/organisms but several studies have demonstrated that both γ-lactone and THP/THF moieties are essential for complex I inhibition [26–28]. Intriguingly, chamuvarinin and B-THP-Ts are toxic to procyclic form (PF) and bloodstream form (BSF) *T*. *brucei* [15–17] with EC_50_ values in the low micromolar range (**Fig 1**), however, complex I is not essential in either form of the parasite [29,30], and our B-THP-Ts lack the terminal γ-lactone indicating that our compounds must have a different mode of action in kinetoplastids. In order to further optimise the potency and selectivity of our trypanocidal B-THP-T compounds this study set out to determine their precise target(s) in *T*. *brucei*.

The identification of targets of lead compounds is often necessary to allow subsequent lead optimisation, but target identification is a major bottleneck in the drug discovery pipeline following phenotypic screens. Photo-affinity labelling provides an elegant solution by covalently attaching a bifunctional photo-affinity probe analogue of the lead compound to the target for its ultimate detection (for review see [31] and **Fig 1A & 1B**) and can readily be achieved by incorporating photoreactive and reporter moieties to the lead compound. Azides, benzophenones and diazirines make excellent photoreactive substituents as they decompose upon UV-irradiation to form short-lived reactive nitrenes, diradicals or carbenes respectively, which rapidly react with neighbouring molecules to form covalent bonds. Hence, by photo-activating the probes once they have been trafficked to their targets, these protein targets can be preferentially labelled. Several radiolabled leads have been functionalised with photoreactive azides [32,33], benzophenones [34] or diazirines [21,35,36] and their targets detected by autoradiograph following protein separation, i.e. SDS-PAGE. A similar, but radiolabel free, methodology has also been employed by functionalising an inhibitor with both a photoreactive diazirine and a fluorescent reporter moieties, allowing the target to be visualised both in gel and within intact cells, thus adding the benefit of target localisation determination. The major drawback of using a radiolabel or fluorescent tags is that, while it allows one to visualise the target, it rarely provides a means to isolate or identify the target. Thus, many [37–44] have used bi-functional photo-affinity probes containing a photoreactive moiety coupled with biotin affinity tag which allows the photo-affinity labelled target to be purified from other proteins using streptavidin beads and identified by tandem mass spectrometry. More versatile yet is the functionalization of the lead compound with photo-affinity group and alkyne handle which allows the subsequent attachment of any desired reporter, such as fluorophore [45–47] or biotin [48,49] using the Cu(II)-catalysed azide-alkyne cycloaddition, also known as the “click reaction” [50] once the photo-affinity probe had been UV-cross-linked to its target.

To identify the target(s) of B-THP-T compounds we synthesised two photo-affinity probe analogues of our lead compound (**Fig 1A**, synthesis to be reported elsewhere): mono-functional **compound 2** contains a photoreactive diazirine, but has no reporter capability; while bi-functional **compound 3** contains the same photoreactive diazirine and an alkyne handle to allow subsequent identification through appendage with biotin affinity or Cy5.5 fluorescent reporter tags. Importantly, the diazirine and alkyne encompass only minor changes to our lead B-THP-T, **compound 1**, and all compounds show similar levels of toxicity (**Fig 1 and S1 Fig**) indicating that the photo-affinity probes work through the same mode of action and are thus suitable for target identification purposes. Here we employed an *in vivo* pulse-chase photo-affinity labelling methodology to covalently attach bi-functional **compound 3** to its target within its biological context and, coupled with liquid chromatography-tandem mass spectrometry identified proteins that are potential targets of B-THP-T compounds. Subsequently, using several metabolic and biochemical assays with lead B-THP-T, **compound 1**, we validated two of those protein hits as binding partners and now allows us to begin work to optimise the potency and selectivity of our inhibitors towards these targets. Furthermore, it demonstrates the strength of this photo-affinity labelling technique, which can be readily applied to any drug discovery program to accelerate the drug discovery process.

## Results and discussion

### Compound 3 photo-affinity labels target(s) specifically

To identify the target(s) of the trypanocidal B-THP-T, **compound 1**, we developed **compound 3**, a bi-functional photo-affinity probe analogue of **compound 1** (**Fig 1A and 1B**, synthesis to be published elsewhere). In addition to the core structure of **compound 1**, **compound 3** contains a diazirine to covalently bind to its target upon UV-activation and an alkyne handle to which an azide reporter, such as a fluorophore or affinity tag can subsequently be added via copper catalysed azide-alkyne cycloaddition, often referred to as the ‘click reaction’ [31,50]. As a negative control, we also developed the mono-functional **compound 2** (**Fig 1A**), which contains the diazirine, but lacks the alkyne and therefore cannot conjugate the azide reporter. Crucially, the addition of the diazirine and alkyne moieties encompass only minor changes to the parent molecule and have minimal effect on overall compound potency (**Fig 1A and S1 Fig**), suggesting that **compounds 1–3** have the same mode of action and that photo-affinity probe **3** is therefore suitable for the identification of target(s) of our B-THP-T compounds.

**Compounds 1–3** were pulse-chased to their targets in live PF *T*. *brucei* cells and UV-irradiated to covalently attach the diazirine-containing compounds to their targets (**Fig 1B**). The pulse-chase methodology was employed rather than labelling in cell lysates so that inhibitors would be trafficked to their target(s) and labelling would therefore occur in its biological context, minimising the likelihood of identifying high affinity targets that do not form *in vivo*. Once the extracted proteins had been subjected to azide-alkyne cycloaddition with Cy5.5-azide, proteins that had been appended with Cy5.5 were detected by SDS-PAGE coupled with fluorescence imaging (**Fig 1C**). As expected, when either the diazirine or alkyne were absent (as in **compounds 1 and 3**) no proteins fluoresced as there was no mechanism for them to covalently bind the Cy5.5 reporter molecule, and this absence of fluorescence indicated that there was minimal non-specific binding of Cy5.5 reporter to cellular proteins. Proteins only incorporated the Cy5.5 reporter tag when diazirine and alkyne were both present on the B-THP-T compound (i.e., with the bi-functional **compound 3**, lane 3 of **Fig 1C**) confirming their essentiality for photo-affinity labelling of protein targets. 18 protein bands were detected to varying degrees, with most prominent bands around 70 and 55 KDa, suggesting major targets of these sizes.

### Protein target(s) are localised to the mitochondria

**Fig 2:**
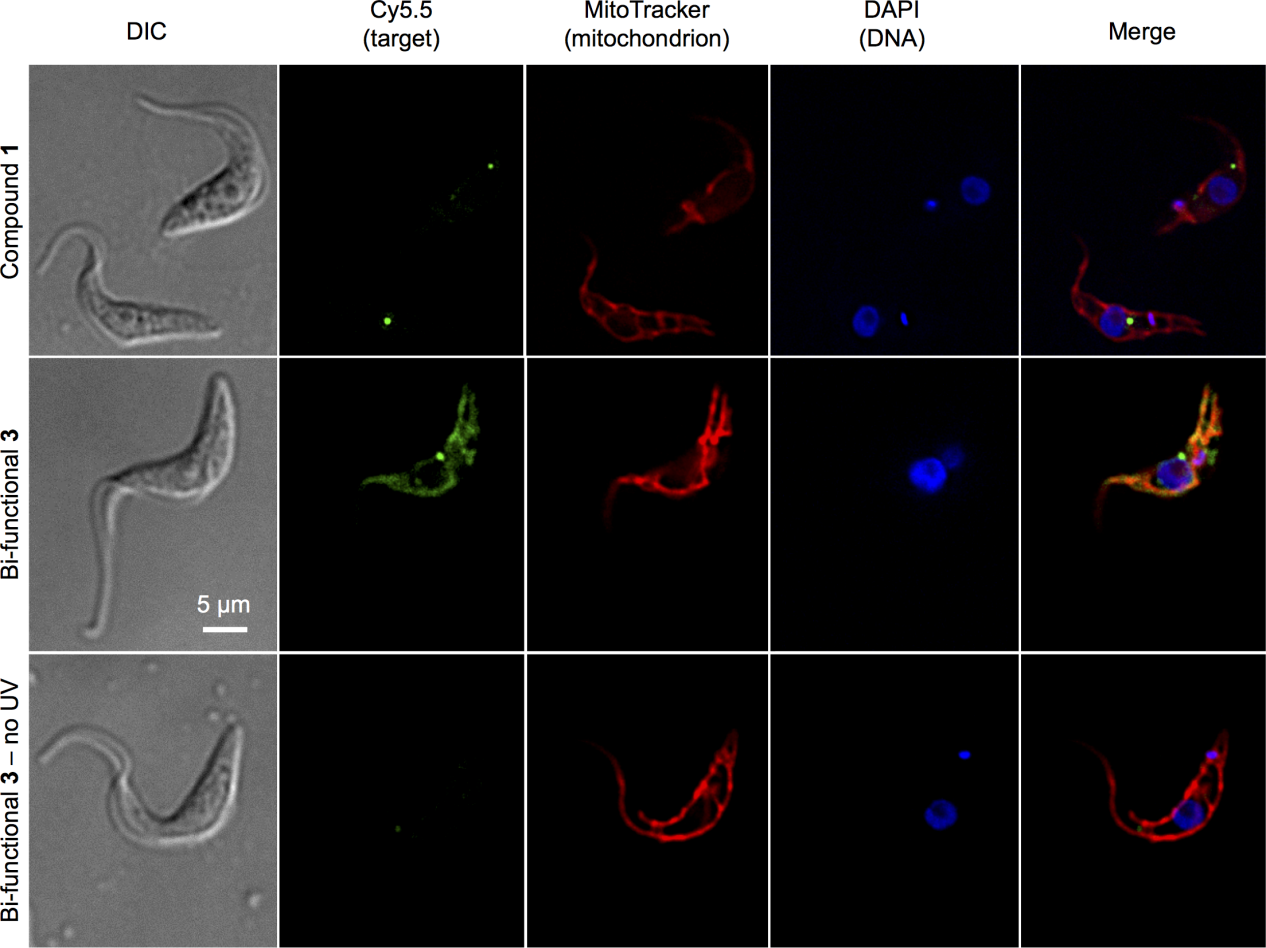
Localisation of B-THP-T target(s). PF *T*. *brucei* cells were *in vivo* pulse-chase photo-affinity labelled with lead B-THP-T **compound 1** or bi-functional **compound 3.** Cells were imaged following in-cell Cy5.5 cycloaddition. Cy5.5 labelling (green) was absent when bi-functional tags were absent (**compound 1**) or when **compound 3** was not UV-activated, indicating that the Cy5.5 labelling observed with UV-activated bi-functional **compound 3** is specific. Cy5.5-labelled proteins co-localise with MitoTracker (red), indicating that B-THP-T compounds target mitochondrial proteins.

To identify the cellular compartments housing B-THP-T protein targets, Cy5.5-azide was “clicked on” to **B-THP-T**-conjugated proteins in fixed whole cells following pulse-chase photo-affinity labelling, and detected by fluorescence microscopy (**Fig 2**). Bi-functional **compound 3** without UV-conjugation and lead **compound 1,** were used as negative controls. As with **Fig 1C**, Cy5.5 labelling only occurred when **compound 3** was cross-linked by UV-activation, again indicating specific labelling of target proteins(s). Cy5.5 labelling was absent from the nucleus, but showed good co-localisation with the MitoTracker staining, suggesting that B-THP-T compounds predominantly target proteins associated with the mitochondria.

### Pull-down hits involve ATP production

**Fig 3:**
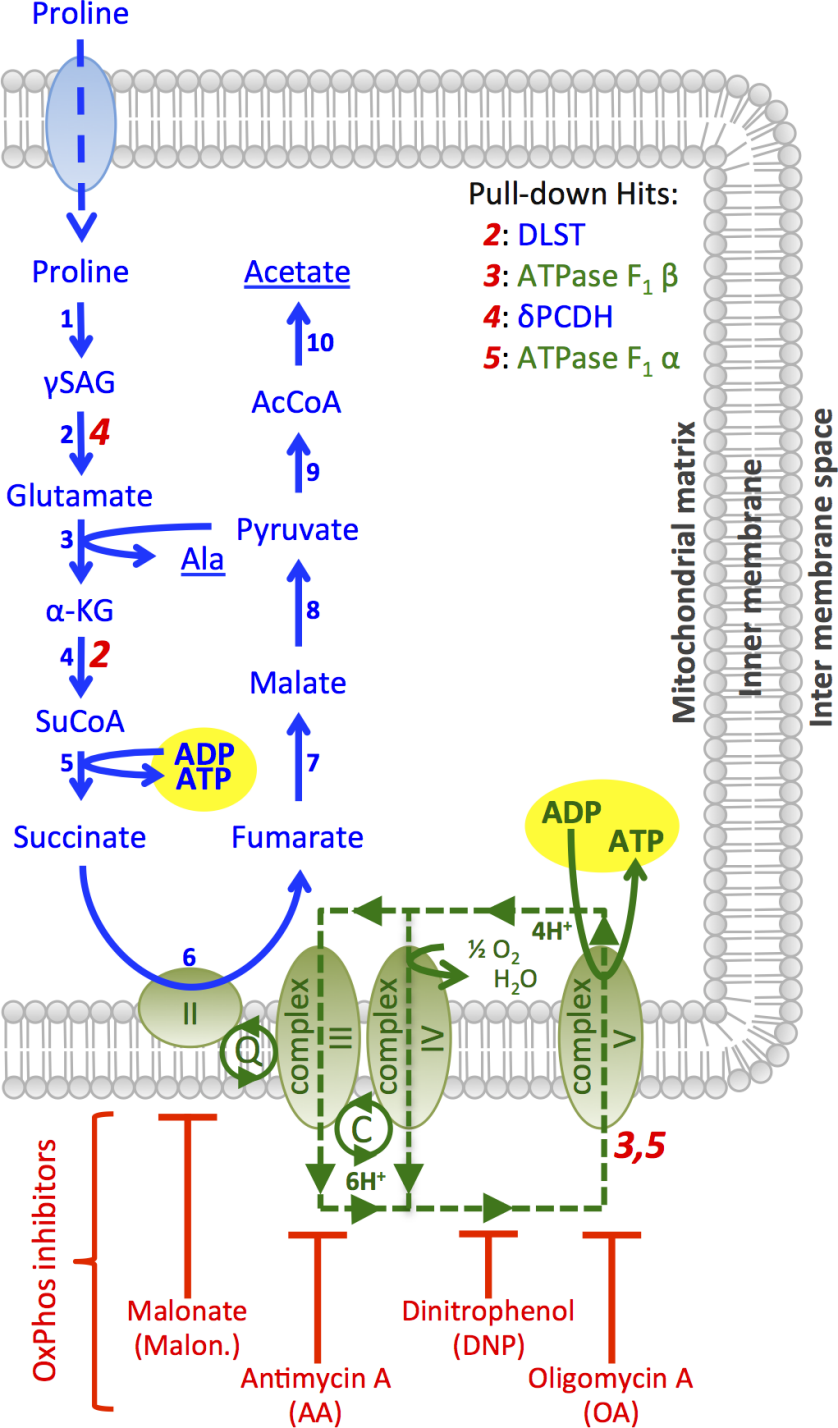
Proline metabolism in PF *T*. *brucei* and reactions catalysed by potential B-THP-T targets. PF *T*. *brucei* grown in glucose-free medium are reliant on proline as a carbon source and enzymes of an incomplete TCA-cycle are used to metabolise it to alanine and actetate (shown in blue): 1, proline dehydrogenase; 2, δpyrroline-5-carboxylate dehydrogenase (δPCDH); 3, L-alanine aminotransferase; 4, a-ketoglutarate dehydrogenase complex (of which dihydrolipoamide succinyltransferase, DLST, is a component); 5, succinyl-CoA synthetase; 6, mitochondrial complex II; 7, fumarase; 8, malate dehydrogenasemalic enzyme; 9, pyruvate dehydrogenase complex; 10, acetate:succinate CoA-transferase. Substrate-level phosphorylation occurs with the conversion of succinyl-CoA to succinate at succinyl-CoA synthetase (step 5). Mitochondrial complex II reduces succinate to fumarate at step 6 and passes electrons into the electron transport chain (represented in green) via ubiquinol (Q). Complex III passes the electrons to cytochrome c (C), and complex IV passes them from cytochrome c to molecular oxygen forming water. Complexes III and IV together export six protons (green dashed line) for each molecule of succinate reduced, and the F_o_F_1_-ATP synthase (complex V) generates ATP by importing four protons, making 1.5 molecules of ATP for every molecule of succinate reduced. The sites of action of oxidative phosphorylation inhibitors are shown. Figure adapted from [19]. Roles of four of the six-mitochondrion pull-down hits are shown in red italic numbers.

Next a series of pull-down experiments were performed to identify the proteins tagged with bi-functional photo-affinity probe, **compound 3**. For pull-down of the target(s), biotin azide was used as reporter and “clicked” on to the B-THP-T-labelled proteins following the same pulse-chase *in vivo* photo-affinity labelling methodology as used above. Tagged proteins were then enriched with streptavidin-agarose, digested *on-bead* with trypsin, analysed by LC-MSMS, and identified through Mascot searches of the *T*. *brucei* proteome. **Compound 1** and mono-functional **compound 2**, which both lacked the alkyne handle required for biotin-azide cycloaddition, were used as negative controls and any proteins pulled down with either of these were eliminated from the **bi-functional compound 3** list of potential protein targets as they represented proteins bound non-specifically to the beads rather than through the biotin-streptavidin interaction. Following elimination of low scoring proteins and unknown proteins (which would be difficult to validate in the timeframe of this study due to their unknown nature), twenty-five *T*. *brucei* proteins were identified as potential targets in three replicate pull-downs from whole cell protein extracts (**S1 Table**). Of those, seventeen (68%) utilised nucleotides as a co-factor, indicating that B-THP-Ts may mimic nucleotides. As localisation studies indicated the target was primarily localised in the mitochondria, attention was focused on the six mitochondrial proteins identified in the pull-down data (**Table 1**). Pleasingly, bands corresponding to proteins of the masses in **Table 1** appear to be Cy5.5-labelled via **compound 3** cross-linking in lane 3 of **Fig 1C**, with the most intense bands (~70 KDa and ~55 KDa) matching closely with the masses of two of the top three hits (72 KDa mitochondrial heat shock protein 70, mHSP70; and 56 KDa ATP synthase F_1_ β-subunit). While this supports the likelihood that the proteins identified in **Table 1** were captured via the streptavidin-biotin/**compound 3** interaction rather than non-specific interaction with the beads, the list only serves as a guide and each of the hits needs to be validated before being identified as a bona-fide target. The most intense Cy5.5-labelled band in **Fig 1C** matched the mass the top-scoring hit in **Table 1** (72 KDa mHSP70), which is under investigation as a B-THP-T target and will be reported elsewhere. Of the remaining five mitochondrial pull-down hits, four (hits 2–5) have roles in the production of ATP through proline metabolism (**Fig 3**) and are the subject of further investigation herein as they can be validated or eliminated through a series of related follow-up assays.

**Table 1:** Mitochondrial proteins identified as potential targets of B-THP-T compounds by biotin pull-down / LC-MSMS.

Hit	Protein	Mass	Nucleotide	Score	Sequences		%
		(KDa)	Cofactor		Tot	Sig	Coverage
1	Mitochondrial heat shock 70 kDa protein (MHSP70)	72	ATP	639	19	16	28
2	Dihydrolipoamide succinyltransferase (DLST)	42	CoA	326	7	7	21
3	ATP synthase F_1_ β-subunit	56	ADP	222	3	3	7
4	δ -1-pyrroline-5-carboxylate dehydrogenase (δPCDH)	63	NAD	207	7	6	14
5	ATP synthase F_1_ α-subunit C-terminal chain	44 *	ADP	158	6	3	13
6	L-threonine 3-dehydrogenase (ThrDH)	37	CoA	148	4	3	12

* In trypanosomatids the F_1_ α-subunit is expressed as a 64 KDa protein and cleaved into a 20 KDa N-terminal and 44 KDa C-terminal chains. Only the 44 KDa C-terminal chain was detected in our pull-downs.

Proline is one of the principle amino acids used as a carbon source for PF *T*. *brucei* in the insect midgut [51,52]. It is taken into the PF mitochondrion and converted to pyruvate through eight enzymatic steps, and then further catabolised to alanine or acetate end products (**Fig 3,** [53]). ATP is produced at two points during this process: first through substrate-level phosphorylation at succinyl-CoA synthetase (**Fig 3,** step 5) as intermediate succinyl-CoA is converted to succinate by succinyl-CoA synthetase; and second through oxidative phosphorylation (the coupling of the electron transport chain (ETC) with ATP production) at the F_o_F_1_-ATP synthase (mitochondrial complex V) as the reduction of succinate to fumarate by mitochondrial complex II feeds electrons into the electron transport chain (ETC). Two of the pull-down hits, δ-pyrroline-5-carboxylate dehydrogenase (δPCDH, hit 4 in **Table 1, Fig 3**) and dihydrolipoamide succinyltransferase (DLST, hit 2 in **Table 1, Fig 3**) have roles prior to substrate-level ATP production, while two other hits, the F_1_ α- and β-subunits (hits 5 and 3 respectively in **Table 1, Fig 3**), form the ADP-binding regulatory and ADP-binding catalytic domains respectively of the F_o_F_1_-ATP synthase and have direct roles in ATP production from oxidative phosphorylation downstream of substrate-level ATP production. Targeting of any of these four hits by B-THP-T compounds would consequently impact cellular ATP production to varying extents. Therefore, a series of biochemical phenotypic studies were undertaken to validate or exclude the pull-down of these four hits.

### B-THP-Ts inhibit cellular ATP production

**Fig 4:**
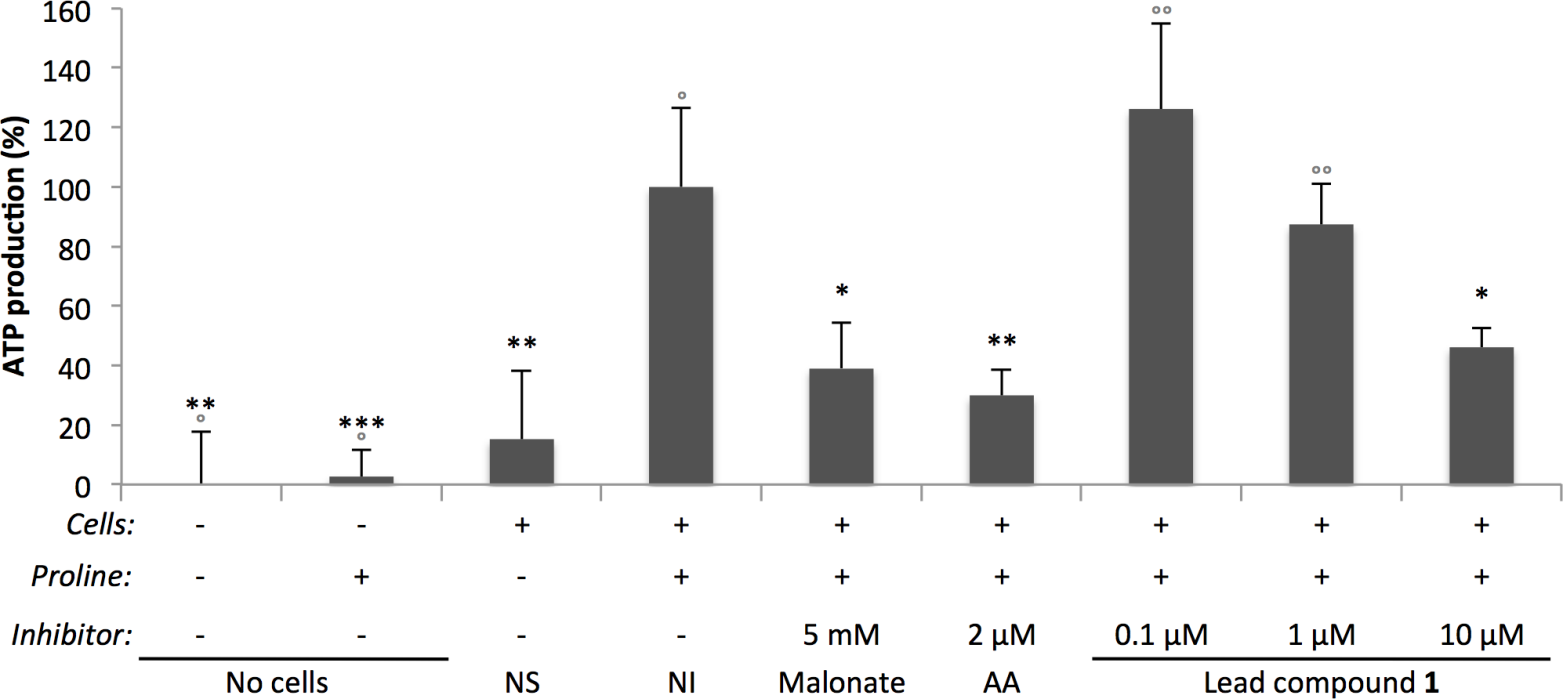
B-THP-Ts decrease cellular ATP levels. PF *T*. *brucei* cells were incubated in buffered PBS with/without proline (as sole carbon source) and various inhibitors (concentrations indicated) incubated for 2 h, after which cells were harvested and cellular ATP levels were monitored by bioluminescence assay. Key: NS, no substrate; NI, no inhibitor control; Malonate (complex II inhibitor) AA, antimycin A (AA, complex III inhibitor). Background relative light units (RLU) were subtracted from measurements and % ATP production normalised to the no inhibitor control. Data were averaged from two independent experiments, each performed in duplicate (n = 4). Error bars show standard deviations. T-tests were performed against no inhibitor controls (black asterisks) and **compound 1** used at 10 μM (grey open circles) and significance values presented: *p* < 0.0005 (*** / °°°), *p* < 0.005 (** / °°), *p* < 0.05 (* / °). The B-THP-T **compound 1** significantly reduces ATP levels in a dose-response manner to levels indistinguishable from known inhibitors of oxidative phosphorylation.

We first set out to determine if B-THP-T compounds affected cellular ATP levels. PF *T*. *brucei* were incubated in buffered PBS with/without proline as the sole carbon source and various inhibitors for 2 h. Cells were harvested and their ATP content was determined by bioluminescence assay. Two ETC inhibitors were used as positive control: malonate is a competitive inhibitor of mitochondrial complex II and prevents the reduction of succinate to fumarate and entry of electrons to the ETC; and antimycin A (AA) is a mitochondrial complex III inhibitor, which prevents the transfer of electrons to cytochrome c and ubiquinone recycling. Both ETC inhibitors significantly inhibited cellular ATP production by 62 ± 15% and 71 ± 9% respectively (**Fig 4**), consistent with them shutting down oxidative phosphorylation but leaving upstream substrate-level phosphorylation at succinyl-CoA synthetase unaffected. **Compound 1** inhibited ATP production in a dose-response manner to similar levels (by 55 ± 6%), supporting the hypothesis from pull-down data that its target is involved in the ATP production pathway from proline.

### B-THP-Ts inhibit ATP production at oxidative phosphorylation

**Fig 5:**
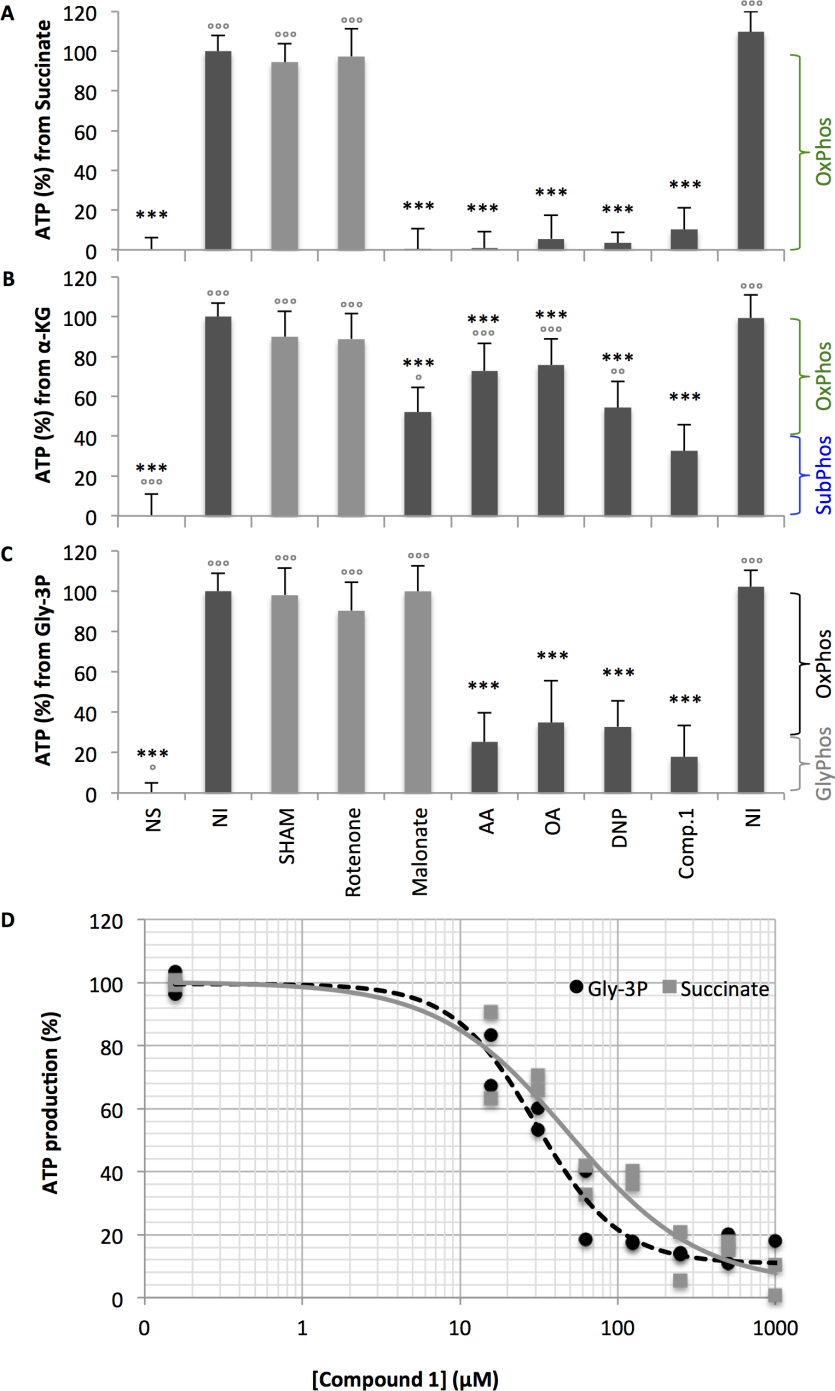
Inhibition of ATP production in digitonin-permeabilsed cells. **(A-C)** Digitonin-permeabilised PF *T*. *brucei* were probed with substrates in the presence of inhibitors and resulting ATP production quantified by bioluminescence assay. See experimental section for details. Inhibitors were used at saturating concentrations to ensure that relevant enzyme activities were ablated. Key: NS, no substrate; NI, no inhibitor; SHAM, salicylhydroxamic acid (TAO inhibitor at 100 μM); Rotenone (complex I inhibitor at 100 μM); Malonate (complex II inhibitor at 5 mM); AA, antimycin A (complex III inhibitor at 200 μM); OA, oligomycin A (complex V inhibitor at 200 μM); DNP, 2,4-dinitrophenol (protonophore at 1 mM); Comp.1, **compound 1** at 200 μM. Background relative light units (RLU) were subtracted from measurements and % ATP production normalised to the no inhibitor control. Data were averaged from three independent experiments, each performed in quadruplicate. Error bars show standard deviations. T-tests were performed against no inhibitor controls (black asterisks) and **compound 1** (grey open circles) and significance values presented: *p* < 0.00005 (*** / °°°), *p* < 0.0005 (** / °°), *p* < 0.005 (* / °). **(A)** Succinate was used as a substrate to generate ATP solely through oxidative phosphorylation (OxPhos). Oxidative phosphorylation inhibitors malonate, AA, OA and DNP virtually eliminated all ATP production. **Compound 1** almost eliminated ATP production and was indistinguishable from inhibitors of oxidative phosphorylation, suggesting that it too inhibits oxidative phosphorylation. **(B)** α-ketoglutarate yields 40% ATP through substrate-level phosphorylation (SubPhos) and 60% through oxidative phosphorylation. Malonate, AA, OA and DNP inhibit oxidative phosphorylation, but not substrate-level phosphorylation. **Compound 1** acted similarly, albeit with a minor additional effect (possibly on DLST, which was also a pull-down hit), suggesting oxidative phosphorylation is its major target. **(C)** Gly-3P acts as an alternative entry site to the electron transport chain yielding ATP through oxidative phosphorylation and the glycosome (GlyPhos). Malonate has no effect, as it acts at a different entry point. AA, OA and DNP inhibit oxidative phosphorylation, but not glycosomal phosphorylation. There is no significant difference between **compound 1** and oxidative phosphorylation inhibitors, suggesting **compound 1** acts downstream of complex II. **(D)** IC_50_ determination of **compound 1** in digitonin-permeabilised cells when succinate (57.3 ± 14.5 μM) or Gly-3P (39.2 ± 4.6 μM) is used as substrate. The efficacy of **compound 1** was similar for both substrates indicating that blockade is downstream of them both.

We next sought to identify the point at which ATP production was blocked using digitonin-permeabilised cells. Digitonin permeabilises the plasma membrane of trypanosomatids, but crucially, leaves the glycosomes and mitochondria intact and fully functional, allowing them to be probed with compartment-specific ATP-yielding substrates [54,55]. Digitonin-permeabilised PF *T*. *brucei* were probed with succinate, which is readily transported across the PF *T*. *brucei* mitochondrial membrane by the dicarboxylic acid carrier [56] and can yield only oxidative phosphorylation-derived ATP through the F_o_F_1_-ATP synthase (complex V). As expected, the mitochondrial complex II inhibitor, malonate, completely eliminated all ATP production from succinate, while downstream inhibitors antimycin A (AA), complex V inhibitor oligomycin A (OA) and protonophore 2,4-dinitrophenol (DNP) almost abolished ATP production (**Fig 5A**). Lead B-THP-T, **compound 1**, decreased ATP production from succinate to similar levels, while rotenone and salicylhydroxamic acid (SHAM), which inhibit the ETC at different entry points and are therefore irrelevant inhibitors, had no significant effect, indicating that **compound 1** inhibits oxidative phosphorylation, and is consistent with the F_1_ α- and/or β-subunits being targets. However, this result on its own does not confirm that the F_1_ subunits are targets, as inhibition of substrate (ADP or succinate) uptake or any of the mitochondrial complexes could have the same effect.

Next, mitochondria were incubated with α-ketoglutarate, which is also taken into the mitochondria by the dicarboxylic acid carrier, to examine the effects of B-THP-T compounds on substrate-level phosphorylation. The multiprotein α-ketoglutarate dehydrogenase complex, of which dihydrolipoamide succinyltransferase (DLST, hit 2 in **Table 1**) is a component, converts α-ketoglutarate into succinyl-CoA (**Fig 3**), allowing succinyl-CoA synthetase to generate substrate-level ATP. In addition, electrons from resulting succinate can enter the ETC to generate oxidative phosphorylation-derived ATP. As complexes III-IV export six protons for every α-ketoglutarate metabolised and the F_o_F_1_-ATP synthase imports only four protons with every ATP synthesised, each mole of α-ketoglutarate can yield 1.5 moles ATP through oxidative phosphorylation. Thus, 40% of the ATP produced from α-ketoglutarate is by substrate-level phosphorylation and 60% is through oxidative phosphorylation. With α-ketoglutarate as substrate, the four oxidative phosphorylation inhibitor controls only blocked up to 48 ± 12% of ATP production (**Fig 5B**) as they were unable to block upstream substrate-level ATP production by succinyl-CoA synthetase. **Compound 1** inhibited ATP production by 67 ± 13%, achieving a significantly lower level of inhibition than that achieved when succinate was used as substrate (**Fig 5A**), suggesting that the blockade is not due to inhibition of substrate uptake, but is due to inhibition of one of the mitochondrial complexes. The greater level of inhibition achieved by **compound 1** over the other oxidative phosphorylation inhibitors suggests that **compound 1** may also be inhibiting substrate-level phosphorylation to some extent, and is, perhaps, an indication that **compound 1** also targets DLST (pull-down hit 2 in **Table 1**). However, the more significant effect of **compound 1** is, by far, on oxidative phosphorylation and the F_1_ subunits are therefore likely to be more significant targets.

Use of the cytosolic oxidative phosphorylation substrate glycerol-3-phosphate (Gly-3P) allowed us to establish whether substrate uptake was a target of our compounds. Mitochondrial Gly-3P dehydrogenase (mGPDH) is located in the intermembrane space of the mitochondrion and converts cytosolic Gly-3P into dihydroxyacetone phosphate (DHAP) while transferring electrons to the ETC via ubiquinone [54]. Gly-3P can therefore be used to probe oxidative phosphorylation at a different entry point from succinate and is not reliant on substrate uptake. When Gly-3P was used as substrate, malonate had no effect on ATP production (**Fig 5C**) as complex II is irrelevant at this ETC entry point. Likewise, other irrelevant inhibitors rotenone and SHAM had no effect on ATP production. However, AA, OA and DNP, which all inhibit oxidative phosphorylation downstream of mGPDH and mitochondrial complex II, reduced ATP production by 75 ± 14%, 65 ± 21% and 67 ± 13% respectively. The inability of oxidative phosphorylation inhibitors to abolish ATP production from Gly-3P has been noted previously [54] and may be the result of glycolytic ATP production following glycosomal uptake of Gly-3P or its product, DHAP. Lead B-THP-T, **compound 1,** acted similarly, inhibiting ATP production by 82 ± 16%. This confirmed that inhibition of oxidative phosphorylation by **compound 1** is not due to inhibition of substrate uptake as Gly-3P is not taken into the mitochondrion and, furthermore, it indicates that the inhibition is downstream of electron entry to the ETC, or more specifically, that **compound 1** inhibits one of complexes III-V.

The potency of lead B-THP-T, **compound 1**, was determined against oxidative phosphorylation in dose-response assays in digitonin-permeabilised PF *T*. *brucei*. The IC_50_ of **compound 1** was 57 ± 15μM and 39 ± 5 μM when succinate and Gly-3P were used respectively (**Fig 5D**). Although these values are higher than in whole cell EC_50_ assays (8.4 μM), it is noteworthy that they cannot be compared directly as the concentrations of proteins and substrates differ greatly between experiments, as do the time-scales of the experiments: digiton-permeabilised cells were plated at over 1000-fold greater density than live cells and used an acute incubation (30 min) rather than the chronic 72 h incubation of the cell viability assay.

### B-THP-Ts block oxidative phosphorylation at the F_o_F_1_-ATP synthase, mitochondrial complex V

**Fig 6:**
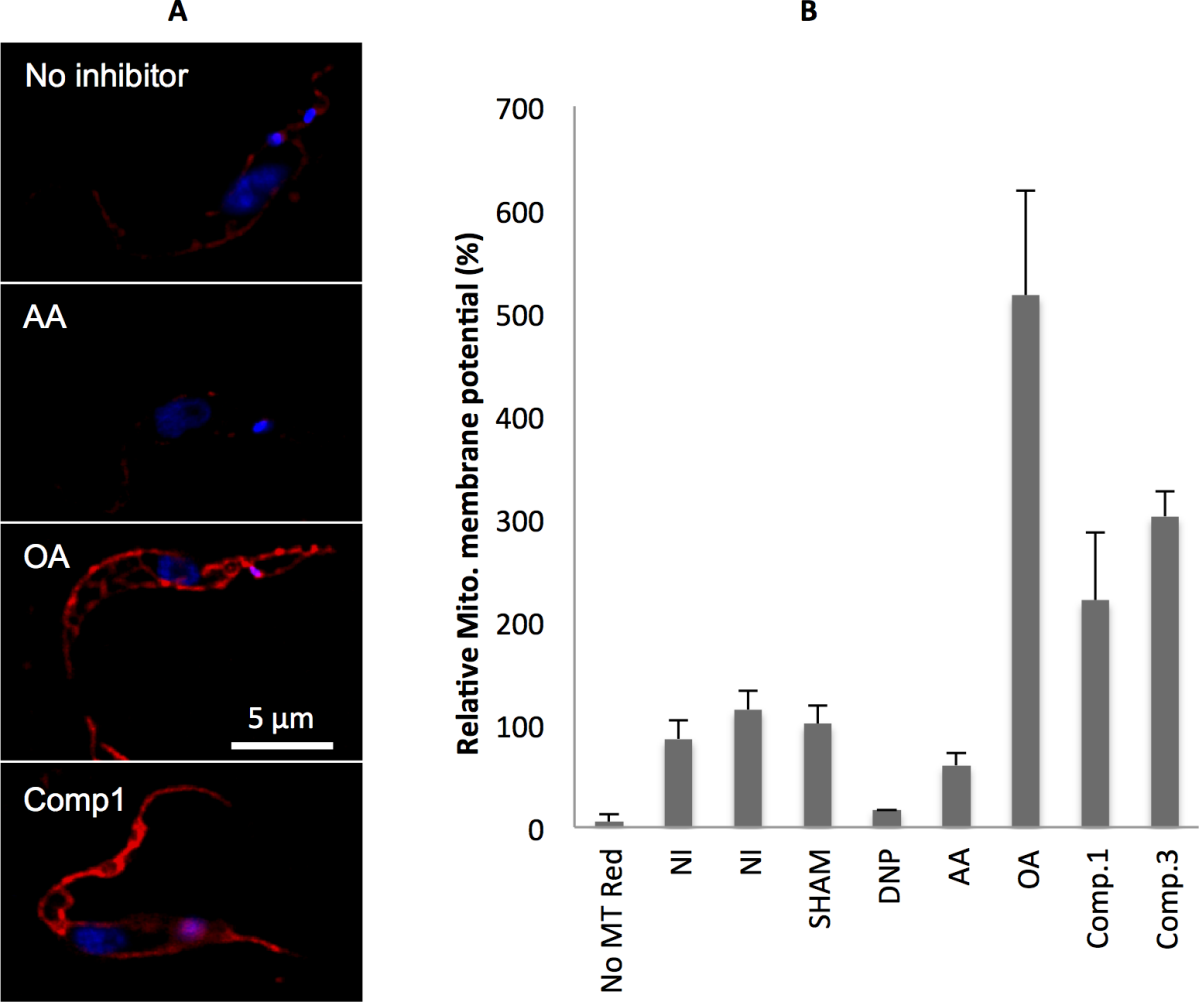
Effects of compounds on mitochondrial membrane potential. PF *T*. *brucei* were incubated with inhibitors and MitoTracker Red CMXRos, which is an indicator of the mitochondrial membrane potential (Δψ_m_). **(A)** MitoTracker-loaded cells were imaged to qualitatively detect differences in the Δψ_m_. Complex III inhibitor, antimycin A (AA at 2 μM) noticeably reduced MitoTracker Red CMXRos uptake, while the F_o_F_1_-ATP synthase (complex V) inhibitor oligomycin A (OA at 2 μM) and **compound 1** at 40 μM clearly enhanced MitoTracker Red CMXRos uptake as compared with the uninhibited control. **(B)** Fluorescence of MitoTracker-loaded cells were quantified using a microplate reader and normalised to MitoTracker green fluorescence. Data were consistent with microscope observations in which inhibitors of the electron transport chain such as Antimycin A (AA at 2 μM), or protonophores such as 2,4-dinitrophenol (DNP at 1 mM) decrease the mitochondrial membrane potential (Δψ_m_), while inhibitors of the F_o_F_1_-ATP synthase such as Oligomycin A (OA at 2 μM) elevate the Δψ_m_. Compounds 1 and 3 at 40 μM elevated the Δψ_m_ indicating that they, like OA, target the F_o_F_1_-ATP synthase.

To determine which mitochondrial complex of oxidative phosphorylation was targeted by B-THP-T compounds, their effects on the mitochondrial membrane potential (Δψ_m_) were monitored in live PF *T*. *brucei* (**Fig 6A**). MitoTracker Red CMXRos, like tetramethylrhodamine and safranine, accumulates in active mitochondria and the degree of accumulation is dependent on the Δψ_m_ [57,58], however, accumulation of MitoTracker Green FM is independent of the Δψ_m_ and the ratio of red/green uptake can therefore be used to determine the Δψ_m_ normalised to the number and size of mitochondria present [59]. In PF *T*. *brucei* mitochondrial complexes III-IV generate a Δψ_m_ by pumping protons out of the mitochondrion during the ETC (**Fig 3**), and thus ETC inhibitors (malonate or AA) prevent proton export and decrease the Δψ_m_ (**Fig 6B**). Conversely, mitochondrial complex V (F_o_F_1_-ATP synthase) uses this potential to generate ATP as it pumps protons back in (**Fig 3**), so inhibition of complex V by OA prevents proton import and elevates the Δψ_m_ (**Fig 6B and** [58]). Similarly, **compounds 1 and 3** elevated the mitochondrial membrane potential (by 2-3-fold), confirming that they, like OA, reduce oxidative phosphorylation by the inhibition of the F_o_F_1_-ATP synthase.

### F_1_ α- and β-subunits are both targeted by B-THP-T compounds

**Fig 7:**
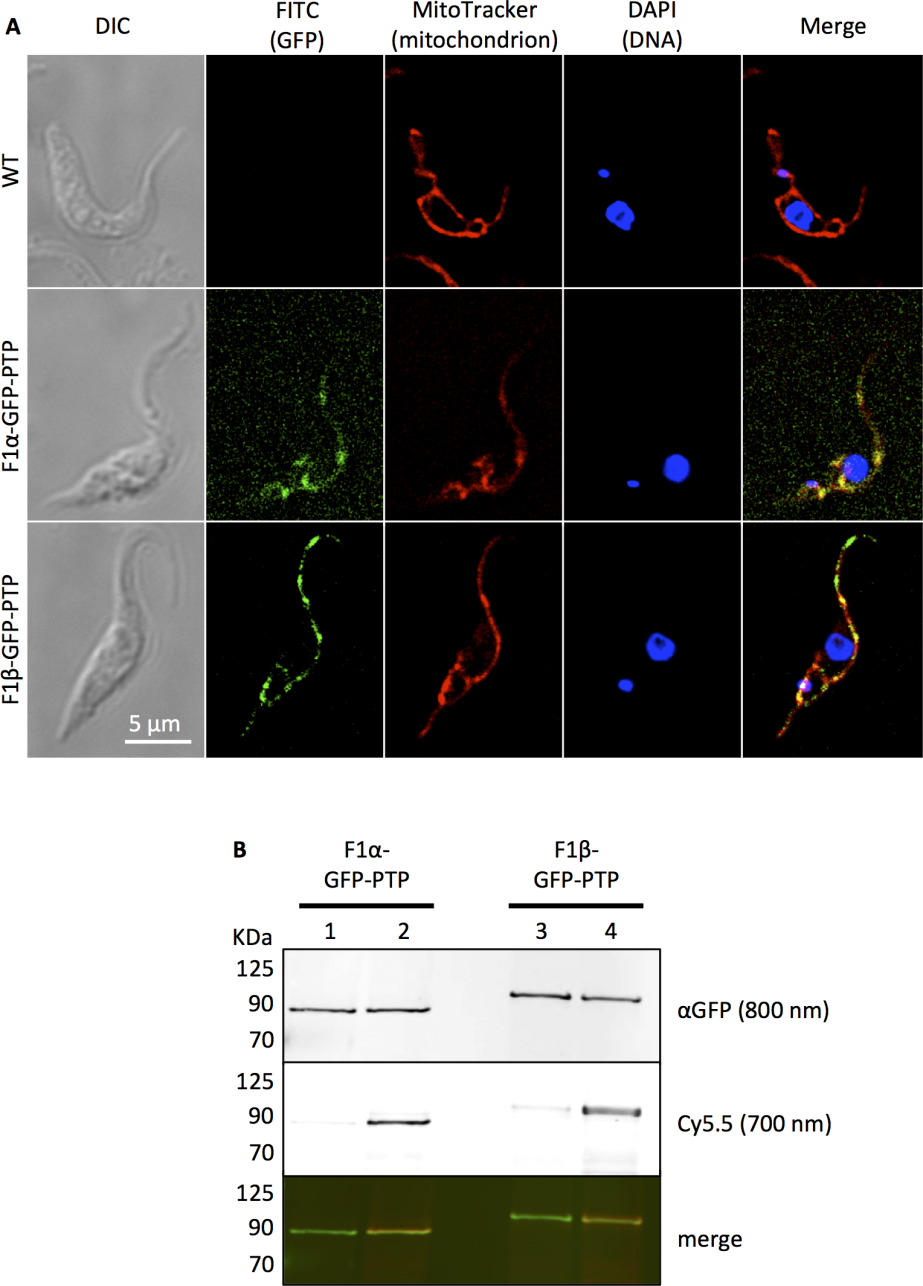
B-THP-Ts bind to C-terminal GFP-PTP tagged F_1_ α- and β- subunits. **(A)** Stably transfected PF *T*. *brucei* cells endogenously-expressing F_1_ α-GFP-PTP or F_1_ β-GFP-PTP were loaded with MitoTracker Red CMXros (red) and DAPI (blue) and imaged microscopically to determine localisation of GFP-PTP-tagged protein. F_1_ α-GFP-PTP and F_1_ β-GFP-PTP were detected through natural fluorescence of their green fluorescent protein (GFP) tag, and both were found to co-localise with MitoTracker, confirming their mitochondrial association. **(B)** Following *in vivo* photo-affinity labelling with 50 μM **compound 3** or **compound 1** (negative control), GFP-PTP-tagged F_1_ α- and β-subunits were affinity purified and Cy5.5 clicked on. Proteins were separated by SDS-PAGE and a western blot was performed with mouse anti-GFP / anti-mouse-DyLight 800. GFP-PTP-tagged proteins were revealed at 800 nm, Cy5.5-bound target proteins at 700 nm, and a merged image shows both together. Lanes 1 and 2 used **compounds 1 and 3** respectively with F_1_ α-GFP-PTP-expressing cells and captured F_1_ α-GFP-PTP migrating at 89 KDa was observed with anti-GFP at 800 nm in both lanes. F_1_ α-GFP-PTP only fluoresced at 700 nm when bi-functional **compound 3** was used, indicating that Cy5.5 had bound specifically to the F_1_ α-subunit via our bi-functional photo-affinity probe. Lanes 3 and 4 used **compounds 1 and 3** respectively with F_1_ β-GFP-PTP-expressing cells, and captured F_1_ β-GFP-PTP migrating at ~101 KDa was observed in both lanes with anti-GFP. As with the α-subunit, β-GFP-PTP only conjugated Cy5.5 when the bi-functional **compound 3** was used, confirming that both the α- and β-subunits are targets of our B-THP-T compounds.

The F_1_ α- and β-subunits of the F_o_F_1_-ATP synthase were both identified as potential B-THP-T targets through biotin pull-down (**Table 1**), and the F_o_F_1_-ATP synthase was shown above to be inhibited by lead B-THP-T **compound 1**, but to confirm whether both, or just one of the F_1_ subunits is a target of our compounds, we generated cell lines endogenously expressing α- or β-subunits with C-terminal GFP-PTP tags. The PTP affinity tag comprises epitopes for proteins C and A separated by a TEV protease cleavage site for efficient purification of the target protein and the cloning methodology facilitates recombination within the endogenous targeted gene rather than exogenous integration, allowing for endogenous levels of expression [60]. The endogenous expression produces tagged protein at physiological levels, allowing it to be trafficked and form complexes in the same way as untagged endogenously expressed protein, as previously demonstrated through the purification of intact F_o_F_1_-ATP synthase with a C-terminally TAP-tagged F_1_ β-subunit [61]. In addition, we cloned green fluorescing protein (GFP) into the tag to act as an additional reporter and for antibody-free localisation studies. Fluorescence imaging (**Fig 7A**) confirms that both tagged subunits correctly localise to the mitochondria.

Pulse-chase *in vivo* photo-affinity labelling with **bi-functional compound 3** (and **lead compound 1** as negative control) was performed on cells from both cell lines expressing GFP-PTP-tagged F_1_ α- or β-subunits. Tagged subunits were immobilised on IgG-sepharose beads, purified, and Cy5.5 fluorescent reporter clicked on, and proteins were detected by western blotting (**Fig 7B**). Mouse anti-GFP coupled with anti-mouse-DyLight-800 identified tagged F_1_ α-GFP-PTP and F_1_ β-GFP-PTP subunits migrating as expected at approximately 89 KDa and 101 KDa respectively. In trypanosomatids (but not in other organisms) the native α-subunit is expressed as a 63 KDa protein which is then cleaved by an unknown protease to 20 KDa N-terminal and 43 KDa C-terminal domains, both of which remain associated with the F_o_F_1_-ATP synthase complex [61,62]. Pleasingly the 109 KDa GFP-PTP C-terminally tagged α-subunit also appears to be cleaved at the same position yielding the 89 KDa GFP-PTP-tagged C-terminal domain of the α-subunit, indicating that it is localised and processed within the cell as the native untagged protein. **Fig 7B** shows that approximately equal amounts of GFP-PTP-tagged protein had been captured between **compound 1/3** pairs and between α- and β-subunits (**Fig 7B**), indicating that α- and β-subunits were expressed (and captured) to similar levels. However, for both α- and β-subunits, Cy5.5 fluorescence at 700 nm was only detected appreciably in samples photo-affinity labelled with bi-functional **compound 3**, and not with **lead compound 1**. This indicates that **compound 3** can UV-conjugate to both F_1_ α- and β-subunits and corroborates their pull-down in Table 1.

### B-THP-Ts model to the F_1_ α- and β-subunit ATP-binding sites

**Fig 8:**
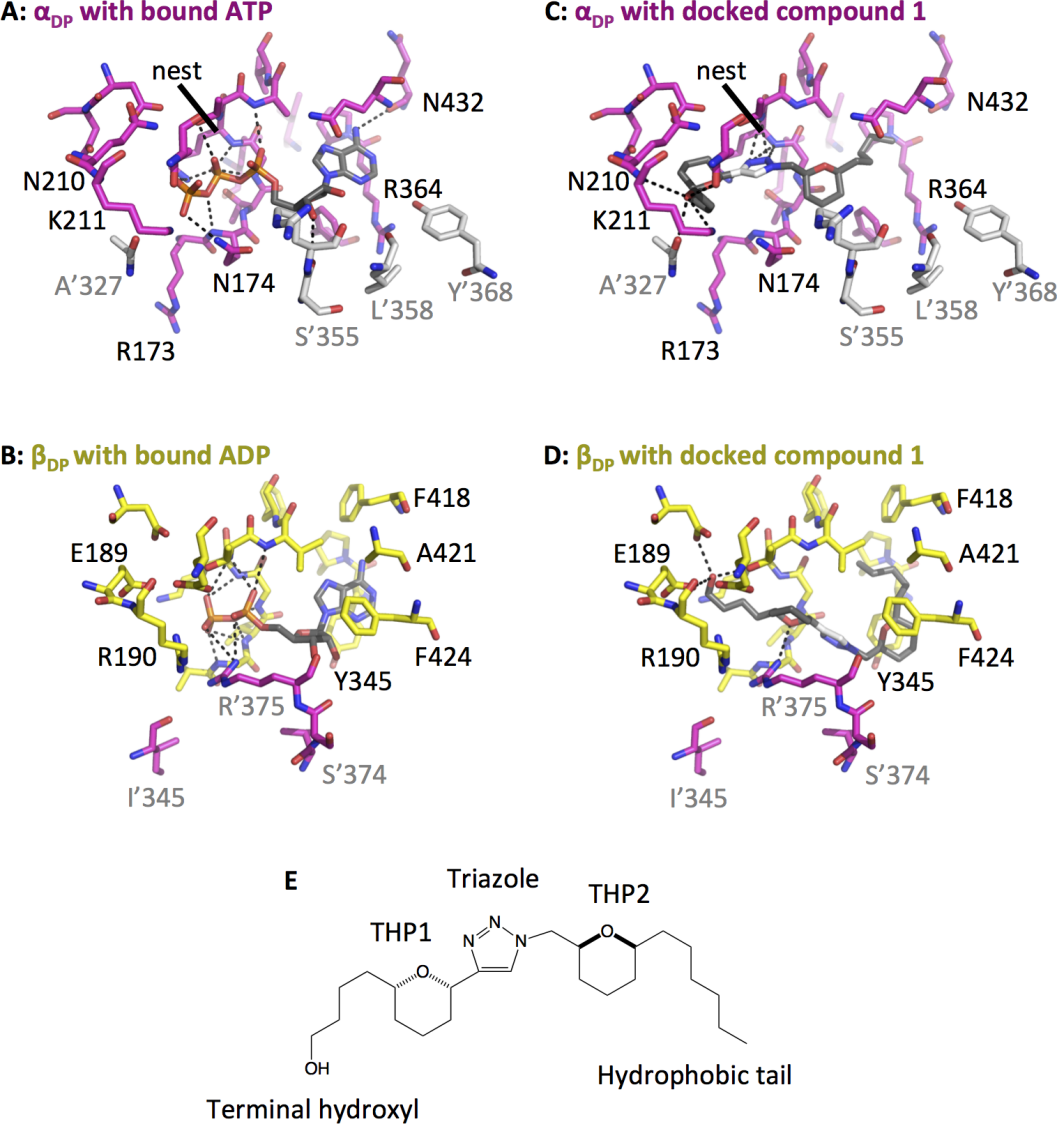
Binding of nucleotide and compound 1 to yeast F_1_ α- and β-subunits. Yeast F_1_ subunits are oriented around the Walker A motif for comparisons. **(A and B)** The crystal structure yeast F_1_ with bound nucleotide (PDB entry 2WPD [68]). ATP occupies that regulatory site of the α-subunit **(A)**, while ADP occupies the catalytic site of the β-subunit **(B)**. The positioning of nucleotide is similar for both: the adenine anchors within a hydrophobic pocket while the phosphates interact with the Walker A nest. Hydrogen bonds are shown as dotted lines. **(C-D)** Docking of **compound 1** into the yeast ATP binding sites. In the regulatory α-subunit **(C)** the triazole moiety of **compound 1** interacts with the Walker A nest, THP2 occupies the position of the nucleotide ribose, the hydrophobic tail buries into the hydrophobic adenine pocket, and the terminal hydroxyl forms extensive H-bonds. The position is **compound 1** is different in β-subunit **(D)**, whereby THP2 is sandwiched between Tyr345 and Phe424, the hydrophobic tail buries into the adenine site, and THP1 and terminal hydroxyl form potential H-bonds. **(E) Compound 1**.

F_1_ of mitochondrial complex V is a multi-protein complex composed of α-, β-, γ-, δ- and ε-subunits with stoichiometry 3:3:1:1:1 [63,64]. The α- and β-subunits form a catalytic heterohexamer of alternating subunits with ADP/ATP binding sites at their interfaces (**S2 Fig** and **Fig 8A and 8B**) while γ- and ε-subunits form an asymmetrical central stalk connected to the F_o_ moiety which together, driven by the proton-motive force, rotate within the α/β hexamer to drive ATP production by introducing conformational changes at the catalytic sites and changing their affinities for ADP and ATP [63,65–67]. X-ray crystal structures of F_1_ have been determined for several species, but no high-resolution structure of trypanosomatid F_1_ have been solved. Consequently, to determine likely B-THP-T binding sites over the entire F_1_ moiety, B-THP-T **compound 1**, which displays only modest selectivity towards *T*. *brucei* over mammalian HeLa cells (**Fig 1**), was docked into the entire F_1_ crystal structures from *Sacharomyces cerevisiae* (PDB entry 2WPD [68]) and *Bos taurus* (PDB entry 1BMF [63]) using AutoDock Vina [69]. For both crystal structures, catalytic and regulatory ATP binding sites were identified as sites of lowest binding energy and therefore most likely binding positions of B-THP-T compounds.

The F_1_ α- and β-subunits are paralogues of one another with sequence identity of 20–25% (value depending on species), and while the β-subunit has catalytic activity, the α-subunit has lost this ability and instead plays a regulatory role in ATP synthesis and hydrolysis. They share a common RecA-like protein fold along with other classes of ATP-binding proteins including kinases, phosphatases, ATPases, heat shock proteins, transfer/transport ATPases, and permeases [70,71], in which the adenine of the nucleotide binds within a sequence-non-specific hydrophobic pocket and the B-phosphate within the amine nest of the conserved Walker A motif (sequence GxxxxGKT/S) (**Fig 8A and 8B**). Although the mode of ATP-binding is similar for α- and β-subunits, their three-dimensional shapes differ due to differences in amino acid sequence. The three ATP-binding regulatory sites of the α-subunits (top panel of **S3A Fig**) are all relatively similar, however, the three ATP-binding catalytic sites of the β-subunits (lower panel of **S3A Fig**) are different as their shapes are dependent on the position of the central stalk (and thus state of bound nucleotide). The most significant differences are in the distances between Tyr345 and Phe424, and in the position of the neighbouring α-subunit which completes the catalytic site. The empty site of the β-subunit (β_E_) is open, while the ATP-bound (β_TP_) and ADP + Pi-bound (β_DP_) conformations are closed and somewhat more similar to one another.

**Compound 1** docked similarly within all three regulatory ATP-binding pockets of the yeast α-subunits (α_E_, α_DP_ and α_TP_, top panel of **S4 Fig** and **Fig 8C**). The triazole H-bonded with the amine-rich phosphate nest, THP2 occupied a similar position to the ATP ribose and hydrophobic tail bound within the hydrophobic adenine-binding pocket. THP1 could potentially form an H-bond via a water bridge, while the terminal hydroxyl could H-bond with Thr214, Lys211 of the α-subunit or Ala327 of the neighbouring β-subunit. Interactions between **compound 1** and the bovine regulatory subunits were similar.

**Compound 1** docked similarly within the nucleotide-bound β-subunits (β_DP_ and β_TP_, lower panel of **S4 Fig** and **Fig 8D**) of yeast F_1_ with THP2 sandwiched between Tyr345 and Phe424 and the hydrophobic tail buried within the hydrophobic adenine-binding pocket lined by V165, F166, P346 and F418. The triazole adopted a similar position to the nucleotide’s ribose, allowing THP1 to sit close to the phosphate-binding Walker A nest and the terminal hydroxyl to form extensive H-bonds with Pi-coordinating Arg190, Glu189, Arg260 and Arg’375 (or Adp256 and Thr164). However, in the open B_E_ conformation, Tyr345 and Phe424 lie too far apart to sandwich THP2 and the triazole instead forms interactions with the Walker A nest and THPs potentially forming H-bonds via water bridges.

Docking of compound 1 to the ATP-binding sites of the α- and β-subunits reveals that compound 1 can form similar interactions as ATP and suggests that B-THP-T compounds mimic ADP, a finding which is mirrored by the pull-down of predominantly nucleotide utilising proteins during target identification (**Table 1**). Intriguingly, only one other protein (phosphoenolpyruvate carboxykinase–a glycosomal protein shown in S1 Table) identified as a potential target during pull-down experiments has the Walker A motif, suggesting that the B-THP-T compounds probably do not target all members of this protein family, but instead bind to specific nucleotide-binding sites.

### Conclusions

Until recently drug discovery efforts focussed on the identification of compounds targeting proteins specific to the target organism with the view that such compounds would have low toxicity to the human hosts. Such studies typically began with screening and lead optimisation against recombinant target protein, but projects often derailed during subsequent cell-based testing when compounds were shown to be equally toxic to mammalian cells or compounds failed to reach their targets within the cells. Phenotypic screening removes all previous bias towards targets and brings into play targets that were previously though undruggable by testing compounds in their context within the cell. However, the major bottleneck in phenotypic screening lies in identifying the target after initial screens, often making it difficult to further improve compound potency and selectivity.

We previously identified **compound 1** as a trypanocidal compound during phenotypic screening and this study has successfully identified its protein target and mode of action using a photo-affinity labelling approach. Using a **compound 1**-like bi-functional photo-affinity probe (**compound 3**), we showed that the target is localised to the mitochondria and biotin pull-down experiments identified the α- and β-subunits of the F_o_F_1_-ATP synthase (mitochondrial complex V) as potential targets. We next used **compound 1** to validate our photo-affinity labelling experiments and showed that **compound 1** inhibits cellular ATP production by blocking oxidative phosphorylation at the F_o_F_1_-ATP synthase. Photo-affinity labelling with bi-functional **compound 3** confirmed that both α- and β-subunits are targeted and modelling suggests that **compound 1** binds at their ATP-binding regulatory and catalytic sites respectively. While it is possible that both sites could be targeted, it must be noted that regulatory and catalytic sites sit at the interfaces between α- and β-subunits and it may therefore be possible for photo-affinity probe compound 3 to covalently bind both of the subunits from either catalytic or regulatory site. Regardless, future investigations can target both sites to tailor new compounds that bind specifically to each, and this study highlights the usefulness of photo-affinity labelling in target identification for structure-based drug discovery.

The overall architecture of the F_o_F_1_-ATP synthase is well conserved among different species. Although the α- and β-subunits of *T*. *brucei* F_1_ share 47% and 67% sequence identity respectively with their mammalian homologues, structural differences exist between them. In particular, the trypanosomatid α-subunit is cleaved into 20 KDa N-terminal and 44 KDa C-terminal chains and the recent low-resolution structure of *T*. *brucei* F_o_F_1_ obtained by cryo-electron tomography [62] suggests that this causes a major shift in the position of the α-subunit within the complex with respect to the β-subunit. Crucially, as the catalytic and regulatory binding sites are positioned at the interfaces between α- and β-subunits this is likely to have an effect on the overall shapes and sizes of those sites and a high-resolution structure is warranted to facilitate future structure-based drug design.

The essentiality of the mitochondrial respiration complexes in human hosts may make the F_o_F_1_-ATP synthase (complex V) appear an unattractive target despite the possibility of significant differences between human and trypanosomatid homologues. However, the mitochondrial complexes are being investigated as targets in other diseases. For example, complex I-targeting acetogenins have shown promise as anti-cancer agents in preclinical studies [25], while complex V-targeting Bedaquiline has also recently been approved for the treatment of tuberculosis [72].

The F_o_F_1_ ATP synthase has been shown to be essential in both PF [61] and BSF [73,74] *T*. *brucei*, although its role in each parasite form differs. In the procyclic form (and in mammalian hosts), it is chiefly involved in oxidative phosphorylation whereby mitochondrial complexes III and IV generate a mitochondrial membrane potential (Δψ_m_) by exporting protons from the mitochondrion during the electron transport chain (ETC), and mitochondrial complex V (the F_o_F_1_ ATP synthase) generates ATP while pumping protons back in. Furthermore, the Δψ_m_ is used for other functions, such as protein trafficking [75] and tRNA import [76]. BSF *T*. *brucei* lacks much of the oxidative phosphorylation machinery (namely, complexes III and IV, [77]) as it generates sufficient ATP through glycolysis [78] and generates the essential Δψ_m_ by running F_o_F_1_-ATP synthase in reverse, hydrolysing ATP instead of producing it [73,79,80]. The difference in function between mammalian and BSF *T*. *brucei* F_o_F_1_ ATP synthase (ATP production versus mitochondrial membrane polarisation respectively) could be exploited for the creation of synergistic drug combinations that do not affect the mammalian hosts.

## Materials and methods

### Cell culture and generation of stably transfected cell lines

PF double marker strain 29–13 were maintained as reported previously [81] in full growth medium (SDM-79 medium supplemented with 10% foetal bovine serum (FBS) (Gibco), 2 g/L sodium bicarbonate, 7.5 mg/L haemin,15 μg/mL neomycin (G418) and 50 μg/mL hygromycin) at 27°C with 5% CO_2_. For experiments using low-glucose growth medium, PFs were adapted to glucose-free SDM-79 supplemented with 10% FBS, 2 g/L sodium bicarbonate, 7.5 mg/L haemin and maintained as above.

#### Subunit-GFP-PTP vector construction

The terminal 735 nucleotides of the α-subunit and β-subunit of complex V were PCR amplified using primers 5’-AATTGGGTACCCCATGGTCACGGCTCCTGGAACGC / 5’- ATCTTCCTTAAGCTCGAGTCTAGACACTGCCCGCTTACAAGAGTAG and 5’-GGTACCCCATGGCAAGATGTGCTTCTTTTTATCGAC / 5’-CTCGAGTCTAGAGCTACTGGCTTGAGCAACACGG respectively, and GFP was PCR amplified using primers 5’- TCTAGACTCGAGAGCAAGGGCGAGGAGCTGTTCACC / 5’-ATCTTCCTTAAGCTTGTACAGCTCGTCCATGCCGAGAGTG. To generate the GFP-PTP-tagged α-subunit, α-subunit and GFP, PCR products were XhoI-digested, ligated together and inserted into the pC-PTP vector [60] using restriction sites KpnI and BamHI. To generate the GFP-PTP-tagged β-subunit, the α-subunit was exchanged for the β-subunit using NcoI and XbaI restriction sites.

#### Transfection and selection of stable cell lines

Cell lines were generated as previously reported [82,83]. Briefly, 10^7^ PF cells (strain 29–13) were transfected with 10 μg MfeI-linearised pC-F1α-GFP-PTP vector or BsmI-linearised pC-F1β-GFP-PTP vector using an Amaxa electroporator and T-cell nucleofector kit (Lonza) as per the manufacturer protocol. Stable transformants were selected in and maintained in full growth medium supplemented with 2 μg/mL puromycin at 27°C with 5% CO_2_.

### B-THP-T pulse-chase photo-affinity labelling

#### B-THP-T compounds

Synthesis of B-THP-T compounds in our laboratory was reported previously: lead **compound 1** (compound 23 in [17]); mono-functional **compound 2**, 4-((5S,7S)-7-((4-((2S,6S)-6-(4-(benzyloxy)butyl)tetrahydro-2H-pyran-2-yl)-1H-1,2,3-triazol-1-yl)methyl)-6-oxa-1,2-diazaspiro[2.5]oct-1-en-5-yl)butan-1-ol (synthesis to be published elsewhere); bi-functional **compound 3**, (4-((5S,7S)-7-((4-((2S,6S)-6-(4-(benzyloxy)butyl)tetrahydro-2H-pyran-2-yl)-1H-1,2,3-triazol-1-yl)methyl)-6-oxa-1,2-diazaspiro[2.5]oct-1-en-5-yl)-N-(prop-2-yn-1-yl)butanamide (synthesis to be published elsewhere).

#### *In vivo* photo-activatable-cross-linking

During the pulse phase of the experiment, WT PF cells were incubated with B-THP-T compound at 50 μM in full growth medium for 1 h. Cells were washed in PBS to remove excess compound and cells were incubated in full growth medium for an additional 30 min to chase uptaken compound to its target. Cells were washed and suspended in PBS, prior to UV irradiation at 365 nm for 10 min at 100 W. Proteins were MeOH/CHCl_3_-precipitated and stored at -20°C until required.

#### Cycloaddition of Cy5.5 and detection

Azide probe was conjugated to alkyne-tagged protein as previously reported [84]. Briefly, Cy5.5 azide (Jena Bioscience) was added to B-THP-T-cross-linked protein using the Click-iT Protein Reaction Buffer Kit (Thermo Fisher Scientific) as per manufacturers protocols. Proteins were separated by SDS-PAGE using standard protocols. Gels were imaged with an Odyssey Imaging System (Li-Cor) and Cy5.5 detected at 700 nm. Images were processed using Li-Cor Image Studio (Version 4.0).

#### B-THP-T cross-linked protein localisation

B-THP-T compounds were pulse-chased and cross-linked to their targets *in vivo* as outlined above, but they were UV-irradiated in full growth medium. Live UV-irradiated WT cells were probed with 100 nM MitoTracker Red CMXRos (Thermo Fisher Scientific) for 20 min and unincorporated dye was washed away. Cells were fixed in 4% paraformaldehyde (PFA) in PBS, adhered to poly-lysine-coated slides and permeabilised with 0.1% TX-100 in PBS for 10 min. Cells were washed copiously with 2% bovine serum albumin (BSA) (Sigma) in PBS. Cy5.5 was added to inhibitor-cross-linked proteins using the Click-iT Cell Reaction Buffer Kit (Thermo Fisher Scientific) as per manufacturers protocols. Unincorporated Cy5.5 azide was removed by extensive washing with 2% bovine serum albumin (BSA) in PBS. Coverslips were mounted with DAPI-containing Slow Fade Diamond Antifade mounting medium (Thermo Fisher Scientific). Images were collected using a Deltavision wide field fluorescent light microscope and processed using SoftWorX software. DAPI was excited at 387 ± 6 nm and detected at 447 ± 30 nm; MitoTracker Red CMXRos was excited at 560 ± 25 nm and detected at 624 ± 20 nm; and Cy5.5 was excited at 650 ± 7 nm and detected at 684 ± 12 nm. Exposure settings were optimised for the sample with **compound 3** and applied to all samples for image consistency.

### Protein target pull-down and identification

#### Biotin pull-downs

B-THP-T compounds were cross-linked to proteins in 5 x 10^7^ WT cells as outlined above and total protein content extracted by MeOH/CHCl_3_-precipitation. **Compound 3** was used as the functional B-THP-T, while **compound 1** or **compound 2** were used as non-reactive negative controls. Cross-linked pellets were solubilised in 1 mL pull-down buffer (PBS, 0.1% SDS) and insoluble material removed by centrifugation. Supernatants were added to 50 μl streptavidin agarose (Sigma) and agitated with end-over-end agitation for 1 h, at RT. Sticky proteins and endogenously biotinylated proteins were removed by centrifugation. The supernatant was MeOH/CHCl_3_-precipitated and biotin azide (Sigma) added using the Click-iT Protein Reaction Buffer Kit (Thermo Fisher Scientific). Proteins were MeOH/CHCl_3_-precipitated to remove free biotin and pellets were resolubilised in 1 mL of pull-down buffer. 20 μl of streptavidin agarose was added and incubated with end-over-end agitation for 1 h at 4°C. Beads were pelleted by centrifugation and washed 4 times in PBS / 0.02% SDS to remove non-bound proteins. Beads were then washed 6 times in 50 mM ammonium bicarbonate to remove SDS for subsequent applications.

#### On-bead tryptic digest

Beads were pelleted and suspended in an equal volume of 10 mM ammonium bicarbonate to create a 50% slurry. Trypsin (0.2 μg) was added and samples were digested overnight at 37°C. Samples were centrifuged for 5 min at 8000 rpm and the peptide-containing supernatants recovered. Peptides were concentrated down to 5 μL with a SpeedVac (ThermoSavant) and made up to 20 μL with loading buffer (98% water, 2% acetonitrile, 0.05% trifluoroacetic acid). For each sample, half (10 μL) was analysed by LC-MSMS (see below).

#### LC-MSMS

Peptides were separated on an Acclaim PepMap 100 C18 trap and an Acclaim PepMap RSLC C18 column (ThermoFisher Scientific), using a nanoLC Ultra 2D plus loading pump and nanoLC as-2 autosampler (Eksigent). Peptides were eluted with a gradient of increasing acetonitrile, containing 0.1% formic acid (5–40% acetonitrile in 15 min, 40–95% in a further 5 min, followed by 95% acetonitrile to clean the column, before re-equilibration to 5% acetonitrile). The eluate was sprayed into a TripleTOF 5600 electrospray tandem mass spectrometer (ABSciex) and analysed in Information Dependent Acquisition (IDA) mode, performing 200 msec of MS followed by 100 msec MSMS analyses on the 15 most intense peaks seen by MS.

#### Protein identification

The MS/MS data files generated were analysed using the Mascot algorithm (Matrix Science) against a database of *T*. *brucei* proteins extracted from NCBI Sept2014 with trypsin as the cleavage enzyme, and 20 ppm peptide mass tolerance. B-THP-T targets were identified by subtracting any proteins pulled down in the negative samples from the list of proteins pulled down with the ‘active’ B-THP-T (**compound 3**). Also removed were proteins with <3 significant peptide identifications, proteins with scores <100, and unnamed proteins. Finally, proteins known not to localise within the *T*. *brucei* mitochondrion were removed from the list of potential B-THP-T targets.

### ATP production assays in digitonin-permeabilised cells

ATP production assays were carried out in duplicate, and three independent assays were conducted. Cells were digitonin-permeabilised as previously described [55]. Briefly, cells were washed in PBS and permeabilised for 5 min at a density of 1x10^8^ cells/mL in SoTE buffer (20 mM Tris.HCl (pH 7.5), 2 mM EDTA pH 7.5, 600 mM sorbitol) supplemented with 0.015% (w/v) digitonin. Permeabilised cells were pelleted at 5000 g for 3 min at 4°C and suspended to 8.4x10^7^ cells/mL in ATP assay buffer (20 mM Tris.HCl (pH 7.4), 15 mM KH_2_PO_4_, 0.6 M sorbitol, 10 mM MgSO_4_, 2.5 mg/mL fatty acid-free BSA.

ATP production assays were carried out in 75 μl volumes in 96-well plates with a final PF *T*. *brucei* cell density of 6.7 x 10^7^ cells/mL as previously reported [55]. Unless otherwise stated, inhibitors were used at high concentration (exceeding their EC_90_ values in live cells) because plating density was >1000-fold higher than with live cell EC_50_ determination and to ensure that the target was fully inhibited. B-THP-Ts, antimycin A (AA) and oligomycin A (OA) were used at 200 μM, Dinitrophenol (DNP) at 1 mM, malonate at 5 mM, rotenone at 100 μM and salicylhydroxamic acid (SHAM) at 100 μM. 59.25 μL digitonin-permeabilised cells (prepared above) were added to 0.75 μl inhibitor dissolved in DMSO (or 0.75 μL DMSO without inhibitor for no inhibitor control) and incubated for 10 min to allow inhibitors to take effect. Substrate was added to 2.5 mM and incubated for 10 min. Reactions were started with the addition of ADP to 60 μM and incubated at RT for 30 min. Reactions were quenched by the addition of 1.75 μL of 60% perchloric acid and the plate was incubated on ice for 30 min. The acid was then neutralised with 11.5 μL of 1 M KOH. ATP production was quantified using ATP bioluminescence assay kit CLS II (Roche). 10–20 μL from each well was transferred in duplicate to a black 96-well plate. Volumes were made to 50 μL with 0.5 M Tris-acetate pH 7.75. 50 μL luciferase was added to each well, mixed, and precise luminescence was recorded with a Spectramax Gemini XPD (Molecular Devices) set to high sensitivity. Background luminescence was subtracted from each well and ATP levels calculated relative to the uninhibited controls.

### ATP monitoring in live PF cells

Effects of compounds on proline metabolism were determined using a methodology adapted from Podolec et al [85]. Briefly, glucose-free-conditioned PF *T*. *brucei* cells were washed into buffered PBS and incubated in 200 μl volumes with inhibitors for 10 min. 3 mM proline was added as the sole carbon source and cells were incubated for 2 h, after which cultures were spiked with 1 mM citric acid (as internal standard) and cells were pelleted. Supernatants containing metabolic end-products and citrate standard were analysed to evaluate effects on proline metabolism (see below), while the ATP content of cells was determined as follows. Cells were resuspended in 75 μL PBS and lysed with the addition of 1.75 μL 60% perchloric acid. Reactions were incubated on ice for 30 min, then neutralised with 11.5 μL 1 M KOH. ATP content was determined as above.

### MitoTracker-monitoring of mitochondrial membrane potential

#### Qualitative analysis

2 x 10^6^ PF *T*. *brucei* cells were incubated in 1 mL GF-SDM-79 growth media supplemented with/without inhibitors for 2 h. B-THP-Ts were used at 40 μM, AA and OA at 2 μM, DNP at 1 mM and SHAM at 100 μM. MitoTracker Red CMXRos and MitoTracker Green FM were added to 100 nM and 1 μM respectively and incubated for a further 10 min. Cells were washed in growth medium and the MitoTracker reagents chased into the mitochondrion for 10 min in fresh growth medium supplemented with inhibitors. Cells were washed again in growth medium to remove unincorporated MitoTracker reagents, pelleted, suspended in 100 μL 4% PFA in PBS, and fixed at RT in the dark for 10 min. Fixed cells were pelleted and suspended in 100 μl PBS. Cells were transferred to poly-lysine-coated slides and mounted with DAPI-containing SlowFade mounting medium (Invitrogen). Fluorescence images were collected with a DeltaVision microscope (as detailed above) to show MitoTracker localisation and comparative intensity.

#### Quantitative analyses

1 mL of cells at 1 x 10^7^ cells/mL were labelled as above. However, instead of fixing cells they were resuspended in 200 μL growth medium and transferred to 96-well plates. Fluorescences were recorded (excitation at 540/35 nm, emission at 590/10 nm for MitoTracker Red CMXRos, and excitation at 485/20 nm, emission at 528/20 nm for MitoTracker Green FM) using a BioTek FLX800 spectrofluorimeter and Gen5 Reader Control 2.0 software, in much the same way as others have recorded previously [86–89]. Background fluorescence values were subtracted from each and MitoTracker Red fluorescences normalised against their MitoTracker Green FM counterparts. Membrane potentials were then calculated relative to the uninhibited controls.

### Purification of GFP-PTP-tagged F_1_ α- and β-subunits

F_1_ α- and β-subunits were purified using a similar procedure to that previously described for TAP-tagged subunits [61]. Briefly, PF *T*. *brucei* cells from 50 mL culture endogenously expressing GFP-PTP-tagged F_1_ subunits were photo-affinity probed with 50 μM **compound 3** or **compound 1** as outlined above, except cells were lysed in IP buffer (50 mM Tris.HCl (pH 7.6), 150 mM NaCl, 1% TX-100) supplemented with ‘Complete’ EDTA-free protease inhibitors (Roche). GFP-PTP-tagged proteins were solubilsed for 1 h with end-over-end agitation at 4°C and insoluble material pelleted at 16000 g for 10 min at 4°C. The supernatant was applied to 20 μL IgG-sepharose (GE Healthcare Life Sciences) and incubated with end-over-end agitation for 1 h at 4°C. Beads were pelleted at 16000 g for 1 min and non-bound material was discarded. Beads were washed 4 times in 1 mL IP buffer to remove contaminating proteins.

#### On-bead cycloaddition of Cy5.5 and detection

Beads were washed 4 times into 50 mM Tris.HCl pH 8.0 and Cy5.5 azide (Jena Bioscience) was conjugated to immobilised B-THP-T-cross-linked GFP-PTP-tagged protein using the Click-iT Protein Reaction Buffer Kit (Thermo Fisher Scientific) as per manufacturers protocols. Immediately following cycloaddition, beads were washed 4 times in IP buffer to remove reactants and proteins were eluted from the beads directly into reducing SDS-PAGE sample buffer. Proteins were separated by SDS-PAGE using standard protocols and transferred to nitrocellulose. Blots were probed with mouse anti-GFP (Roche) and anti-mouse-DyLight-800 (Thermo) and imaged with an Odyssey Imaging System (Li-Cor). Cy5.5-conjugated proteins were detected at 700 nm, while GFP-tagged proteins were detected at 800 nm. Images were processed using Li-Cor Image Studio (Version 4.0).

### *In silico* docking of compound 1 to F_1_

**Compound 1** was docked into F_1_ from *Bos taurus* (PBD entry 1BMF [63]) and *Saccharomyces cerevisiae* (PDB entry 2WPD [68]) using AutoDock Tools [90] and AutoDock Vina [69] as per the user manuals. Briefly, the energy-minimised 3-dimensional coordinates of **compound 1** were generated with ChemBio 3D (Perkin Elmer) and converted to PDBQT format with AutoDockTools. All non-protein atoms (e.g., waters, nucleotides and metals) were removed from the F_1_ PDB coordinates and hydrogens were added with AutoDockTools. Initially **compound 1** was docked into the entire F_1_ complex using a grid-box of dimensions 126 x 126 x 126 Å containing the entire complex and an exhaustiveness of 48 to account for the large search area. **Compound 1** was next docked into the ATP-binding sites of each α- and β-subunit using a grid-box of dimensions 17 x 15 x 23 Å covering the binding site. Positions of docked compound 1 were evaluated using Pymol (Schrodinger).

## Supporting information

S1 Fig Dose-response of B-THP-T compounds in PF *T*. *brucei*. EC_50_ values were determined for **compounds 1–3** in PF *T*. *brucei*. Compounds had similar potencies, indicating that the tags for photo-affinity labelling have no detrimental effect.
(TIFF)

S2 Fig Architecture of the F_o_F_1_-ATP synthase. The crystal structure of ATP synthase from yeast (PDB entry 2WPD [68]) is shown.
**(A)** α- and β-subunits form a heterohexameric catalytic domain. Driven by the protonmotive force, the asymmetrical γ/δ/ε stalk and F_o_ domains rotate within the catalytic domain, providing mechanical power to generate ATP.
**(B)** Cross-section of the α/β hexamer. ATP binding sites at the subunit interfaces. ATP in regulatory sites is coloured magenta, ADP in catalytic sites is coloured purple.
(TIFF)

S3 Fig Structures of the ATP-binding sites of yeast F1 subunits. Yeast F_1_ subunits, oriented around the Walker A motif, with bound nucleotide. The position of the nucleotide is similar for each subunit despite sequence differences between α- and β-subunits. Large shifts in the position of adjacent subunits can be seen in the catalytic sites (lower images), where R’375 and S’374 move in to close the active site around the bound nucleotide. The regulatory site (upper images) remains relatively unchanged during catalysis.
(TIFF)

S4 Fig Structures of the ATP-binding sites of yeast F1 subunits docked with compound 1. Yeast F_1_ subunits, oriented around the Walker A motif, with docked **compound 1**. **Compound 1** docks similarly into each of the α-subunit regulatory sites, which are all similar in structure. The triazole moiety interacts with the Walker A nest, THP2 occupies the position of the nucleotide ribose, hydrophobic tail buries into the hydrophobic adenine pocket, and the terminal hydroxyl forms extensive H-bonds. The position is different in β-subunits. In the nucleotide-bound sites THP2 is sandwiched between Tyr345 and Phe424, the hydrophobic tail buries into the adenine site, and THP1 and terminal hydroxyl form potential H-bonds. In the open catalytic site of βE Tyr345 and Phe424 lie too far apart to hold THP2 and **compound 1** adopts a different position.
(TIFF)

S1 Table Proteins identified as potential targets of B-THP-T compounds by biotin pull-down / LC-MSMS—complete list. ^a^ Predicted location: C, cytosolic; G, glycosomal; M, mitochondrial; F, flagellar.
(DOCX)
