# Supplementary figures and images for: Photo-affinity labelling and biochemical analyses identify the target of trypanocidal simplified natural product analogues
Source: PLoS Negl Trop Dis. 2017 Sep 5;11(9):e0005886. doi: 10.1371/journal.pntd.0005886 (PMC5608556; doi:10.1371/journal.pntd.0005886)

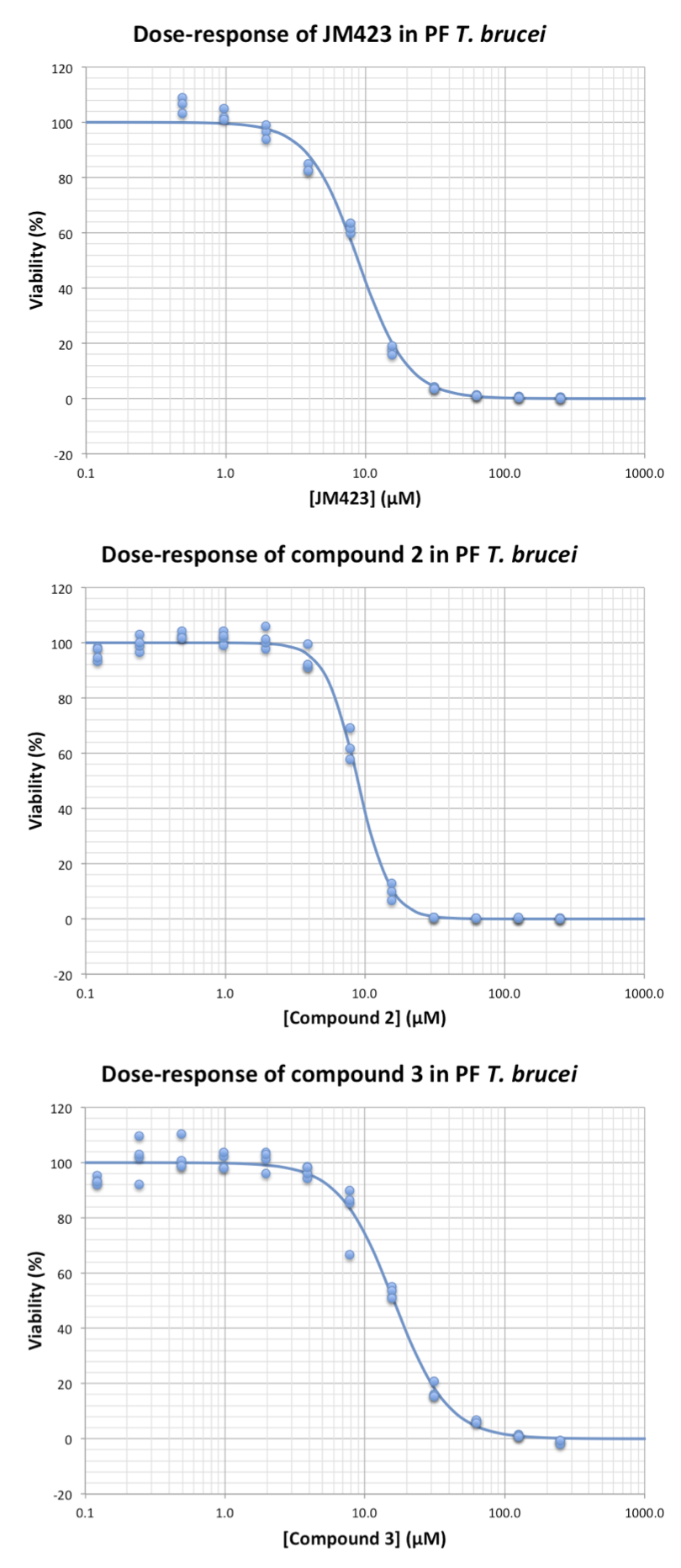

Supplement: S1 Fig — EC50 values were determined for compounds 1–3 in PF T. brucei. Compounds had similar potencies, indicating that the tags for photo-affinity labelling have no detrimental effect. (TIFF) [file pntd.0005886.s001.tiff]

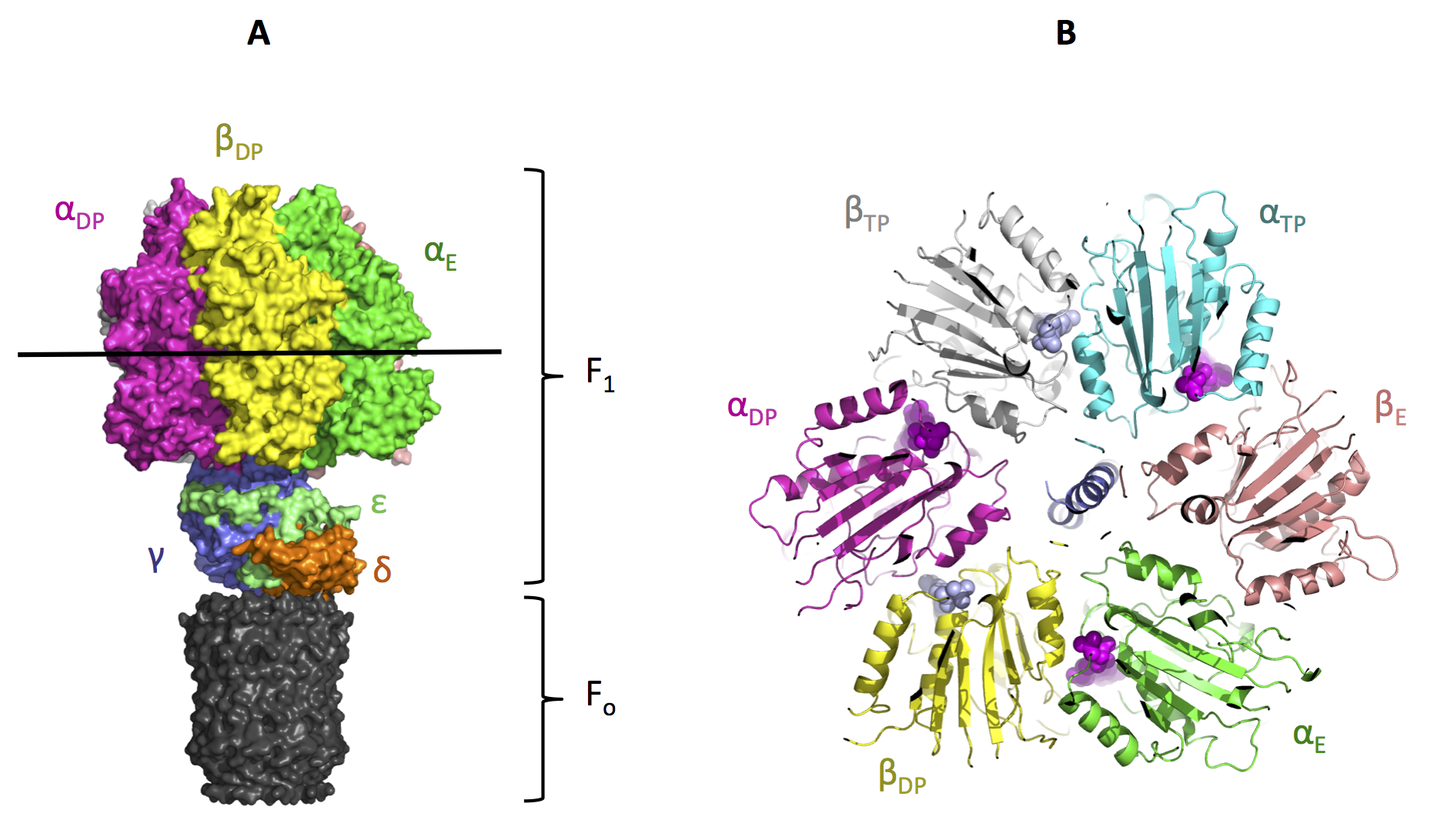

Supplement: S2 Fig — The crystal structure of ATP synthase from yeast (PDB entry 2WPD [68]) is shown. (A) α- and β-subunits form a heterohexameric catalytic domain. Driven by the protonmotive force, the asymmetrical γ/δ/ε stalk and Fo domains rotate within the catalytic domain, providing mechanical power to generate ATP. (B) Cross-section of the α/β hexamer. ATP binding sites at the subunit interfaces. ATP in regulatory sites is coloured magenta, ADP in catalytic sites is coloured purple. (TIFF) [file pntd.0005886.s002.tiff]

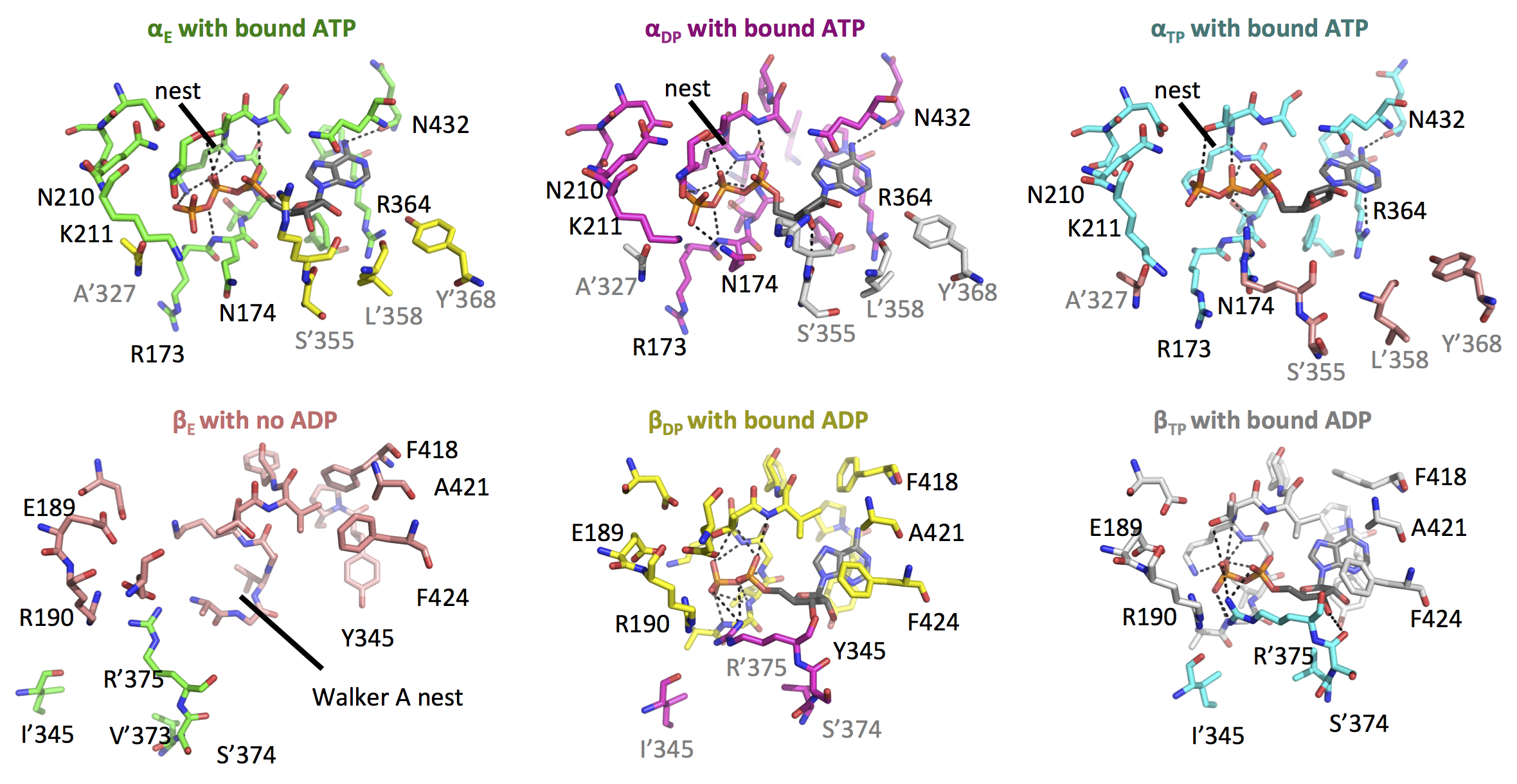

Supplement: S3 Fig — Yeast F1 subunits, oriented around the Walker A motif, with bound nucleotide. The position of the nucleotide is similar for each subunit despite sequence differences between α- and β-subunits. Large shifts in the position of adjacent subunits can be seen in the catalytic sites (lower images), where R’375 and S’374 move in to close the active site around the bound nucleotide. The regulatory site (upper images) remains relatively unchanged during catalysis. (TIFF) [file pntd.0005886.s003.tiff]

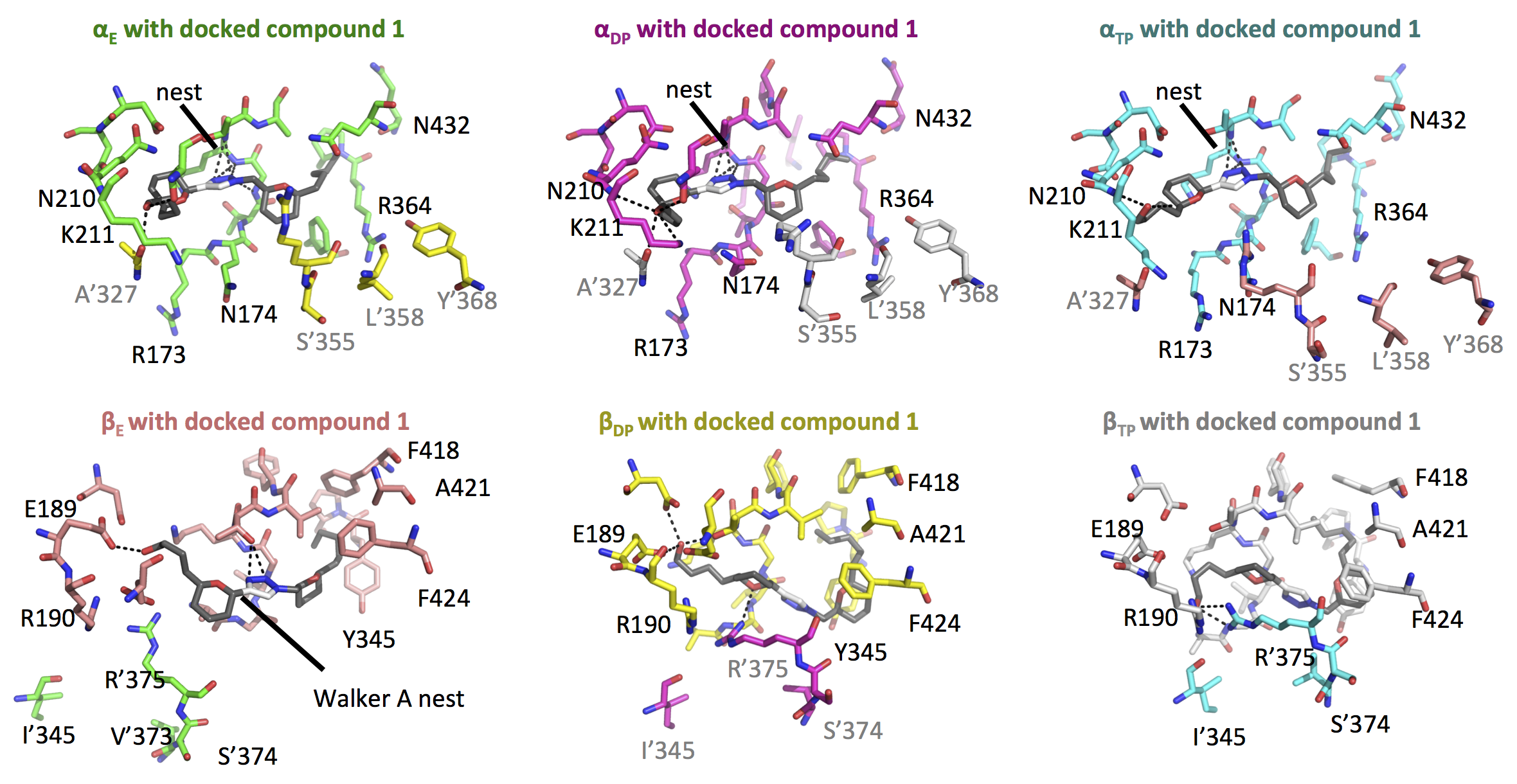

Supplement: S4 Fig — Yeast F1 subunits, oriented around the Walker A motif, with docked compound 1. Compound 1 docks similarly into each of the α-subunit regulatory sites, which are all similar in structure. The triazole moiety interacts with the Walker A nest, THP2 occupies the position of the nucleotide ribose, hydrophobic tail buries into the hydrophobic adenine pocket, and the terminal hydroxyl forms extensive H-bonds. The position is different in β-subunits. In the nucleotide-bound sites THP2 is sandwiched between Tyr345 and Phe424, the hydrophobic tail buries into the adenine site, and THP1 and terminal hydroxyl form potential H-bonds. In the open catalytic site of βE Tyr345 and Phe424 lie too far apart to hold THP2 and compound 1 adopts a different position. (TIFF) [file pntd.0005886.s004.tiff]
