# Supplementary material for: Photo-affinity labelling and biochemical analyses identify the target of trypanocidal simplified natural product analogues
Source: PLoS Negl Trop Dis. 2017 Sep 5;11(9):e0005886. doi: 10.1371/journal.pntd.0005886 (PMC5608556; doi:10.1371/journal.pntd.0005886)
Supplement: S1 Table — a Predicted location: C, cytosolic; G, glycosomal; M, mitochondrial; F, flagellar. (DOCX) [file pntd.0005886.s005.docx]

| **Hit** | **Protein** | **Mass** | **Predicted** | **Nucleotide** | **Score** | **Sequences** | | **%** |
| --- | --- | --- | --- | --- | --- | --- | --- | --- |
|  |  |  | **Location ^a^** | **Cofactor** |  | **Tot** | **Sig** | **Coverage** |
| 1 | Heat shock 70 kDa protein 4 | 71676 | C | ATP | 1232 | 23 | 20 | 38 |
| 2 | enolase | 47133 | G |  | 794 | 7 | 7 | 19 |
| 3 | heat shock protein 83 | 81169 | C | ATP | 720 | 16 | 13 | 23 |
| 4 | 60S ribosomal protein L13a | 39324 | C |  | 714 | 9 | 8 | 20 |
| 5 | mitochondrial heat shock 70 kDa protein | 72000 | M | ATP | 639 | 19 | 16 | 28 |
| 6 | glycerol-3-phosphate dehydrogenase (NAD+) | 38386 | G | NAD | 632 | 6 | 6 | 15 |
| 7 | fructose-bisphosphate aldolase | 41643 | G |  | 528 | 11 | 9 | 29 |
| 8 | S-adenosylhomocysteine hydrolase | 49101 | C | NAD | 392 | 9 | 9 | 17 |
| 9 | pyruvate phosphate dikinase | 101256 | G | ATP | 330 | 9 | 9 | 10 |
| 10 | dihydrolipoamide succinyltransferase | 41516 | M | CoA | 326 | 7 | 7 | 21 |
| 11 | phosphoenolpyruvate carboxykinase (ATP) | 58927 | G | ATP | 283 | 10 | 8 | 20 |
| 12 | tryparedoxin | 15995 | C |  | 258 | 6 | 6 | 47 |
| 13 | arginine kinase | 44955 | G | ATP | 241 | 8 | 6 | 25 |
| 14 | ATP synthase F_1_ β-subunit | 55837 | M | ADP | 222 | 3 | 3 | 7 |
| 15 | δ -1-pyrroline-5-carboxylate dehydrogenase | 62624 | M | NAD | 207 | 7 | 6 | 14 |
| 16 | paraflagellar rod protein | 69953 | F |  | 182 | 5 | 3 | 9 |
| 17 | glycosomal malate dehydrogenase | 33917 | G | NAD | 171 | 4 | 4 | 10 |
| 18 | triosephosphate isomerase | 26989 | G |  | 161 | 6 | 6 | 28 |
| 19 | 73 kDa paraflagellar rod protein | 69096 | F |  | 160 | 8 | 4 | 12 |
| 20 | ATP synthase F_1_ α-subunit | 63862 | M | ADP | 158 | 6 | 3 | 9 |
| 21 | poly(A) binding protein I | 62335 | C | Adenine | 149 | 4 | 3 | 8 |
| 22 | L-threonine 3-dehydrogenase | 37260 | M | CoA | 148 | 4 | 3 | 12 |
| 23 | 6-phospho-1-fructokinase | 53997 | G | ATP | 125 | 5 | 4 | 10 |
| 24 | prostaglandin F synthase | 31201 | C | NADP | 121 | 5 | 3 | 21 |
| 25 | 60S ribosomal protein L9 | 21901 | C |  | 111 | 4 | 3 | 29 |

S1 Table. Proteins identified as potential targets of B-THP-T compounds by biotin pull-down / LC-MSMS - complete list

^a^ Predicted location: C, cytosolic; G, glycosomal; M, mitochondrial; F, flagellar.
